# Language influences music harmony perception: effects of shared syntactic integration resources beyond attention

**DOI:** 10.1098/rsos.150685

**Published:** 2016-02-03

**Authors:** Richard Kunert, Roel M. Willems, Peter Hagoort

**Affiliations:** 1 Max Planck Institute for Psycholinguistics, PO Box 310, 6500 AH Nijmegen, The Netherlands; 2 Donders Institute for Brain, Cognition and Behavior, Radboud University Nijmegen, PO Box 9010, 6500 GL Nijmegen, The Netherlands

**Keywords:** music cognition, syntactic processing, semantic processing, harmony perception, closure ratings, dynamic attending theory

## Abstract

Many studies have revealed shared music–language processing resources by finding an influence of music harmony manipulations on concurrent language processing. However, the nature of the shared resources has remained ambiguous. They have been argued to be syntax specific and thus due to shared syntactic integration resources. An alternative view regards them as related to general attention and, thus, not specific to syntax. The present experiments evaluated these accounts by investigating the influence of language on music. Participants were asked to provide closure judgements on harmonic sequences in order to assess the appropriateness of sequence endings. At the same time participants read syntactic garden-path sentences. Closure judgements revealed a change in harmonic processing as the result of reading a syntactically challenging word. We found no influence of an arithmetic control manipulation (experiment 1) or semantic garden-path sentences (experiment 2). Our results provide behavioural evidence for a specific influence of linguistic syntax processing on musical harmony judgements. A closer look reveals that the shared resources appear to be needed to hold a harmonic key online in some form of syntactic working memory or unification workspace related to the integration of chords and words. Overall, our results support the syntax specificity of shared music–language processing resources.

## 1. Introduction

### 1.1 Syntax versus attention explanations for music–language interactions

Music and language are universal human faculties [1]. Moreover, both of these cognitive domains are based on structured auditory sequences, i.e. they contain discrete elements (e.g. words in language, tones/chords in music) which relate to each other in a rule-governed way in order to form higher order structures (e.g. sentences in language and harmonic sequences in music). This similarity has led researchers to hypothesize that the same processing resources underlie both domains. Supporting evidence comes from a host of experiments which found music harmony manipulations to change language syntax processing [2–8]. However, the nature of the shared resources which lead to these music–language interactions has remained controversial. On the one hand, they are argued to be specific to music and language *syntax*, i.e. they are the result of shared syntactic processing resources [9]. On the other hand, these effects are thought to be an instance of many other, similar, interactions including those between music harmony and language *semantics* [10,11]. In this view, music–language interactions arise due to the effect of music manipulations on general attention. In this paper, a previously little explored direction of influence will be investigated: the linguistic influence on music harmony perception. By doing so, we directly test predictions from a syntax account versus those from general attention accounts.

### 1.2 Music–language interactions as explained by a syntax account

Patel’s [9] shared syntactic integration resource hypothesis (SSIRH) argues that music and language share structural processing resources. It was designed to reconcile neuropsychological evidence for domain independence [12] with neuroimaging studies which found evidence for shared resources in terms of similar activation sites and time-courses for linguistic syntactic processing and tonal harmonic processing^1^ [13,14]. Both kinds of findings together were thought to support a distinction between representations in long-term memory (domain specific, explaining neuropsychological double-dissociations) and resources for online syntactic integration (domain general). Similar activation effects reflect domain-general syntactic integration resources which in turn draw on domain-specific representations.

A key prediction of Patel’s [9] SSIRH is an interaction between music and language syntax processing when both occur at the same time. This prediction has been supported by two behavioural studies finding impaired syntactic integration abilities in language if a concurrent musical tone or chord is difficult to harmonically integrate [2,5]; see also [3,7,8]. For example, Slevc *et al*. [5] presented participants with syntactic garden-path sentences of the form below.

$\text{(a) After}|\text{the trial}|\text{the attorney}|\text{advised}|\text{the defendant}|\underline{\text{was}}|\text{likely}|\text{to commit}|\text{more crimes.}$
$\text{(b) After}|\text{the trial}|\text{the attorney}|\text{advised that}|\text{the defendant}|\underline{\text{was}}|\text{likely}|\text{to commit}|\text{more crimes.}$

Participants reading the sentence in (a) are likely to, at first, misinterpret ‘**the defendant**’ as being advised. Upon encountering the verb ‘was’, readers realize that someone is being advised about ‘**the defendant**’, a syntactic reanalysis which taxes integration resources. In (b) the correct reading is enforced early on when reading ‘that’. As a consequence, reading times on disambiguating words such as ‘was’ are typically longer in sentences like (a) compared with (b). Crucially, Slevc *et al.* [5] found that this syntactic garden-path effect is intensified by a harmonically unexpected (out-of-key) chord presented concurrently with the disambiguating word (‘was’). A timbral unexpectancy (an unusual instrument) was without effect. The authors interpreted this as evidence against acoustic deviancy alone underlying the musical influence on language. The SSIRH explains this pattern by assuming that an out-of-key chord is difficult to integrate into the prevailing key, i.e. it taxes resources involved in syntactic integration. This leaves fewer resources available for concurrently reanalysing the syntactic structure of the sentence, hence the increased linguistic garden-path effect. An unexpected instrument, on the other hand, does not tax integration resources, and hence has no effect.

### 1.3 Music–language interactions as explained by attention accounts

An alternative explanation for the influence of music on reading times is based on general attention mechanisms. These can take two forms. One proposal is an attentional load account. For example, Perruchet & Poulin-Charonnat [11] found a *semantic* garden-path effect when a harmonically unexpected (out-of-key) chord was presented concurrently with the disambiguating word. No semantic garden-path effect was found when the chord was expected (in-key) instead. They found no influence of the music harmony manipulation on semantic *error* processing. The results were argued to support an attentional load account, i.e. one domain (e.g. music) influences another (e.g. language) only if enough attentional resources are left to process both. Semantic garden-path processing is thought to allow for the distribution of attention across domains, while semantic error processing does not. Crucially, the key prediction of this account for the present investigation is that the nature of the processing difficulty, e.g. language syntax or something else, is irrelevant, placing it in sharp contrast to the syntax account by Patel [9].

A different attentional account was put forward by Poulin-Charronnat *et al.* [10] to explain an effect of music harmony on the semantic priming effect seen in lexical decisions. Participants were presented with sung sentences ending either on a word or a non-word and participants were asked to decide on the lexical status of the final item in the sentence. Words could be either semantically expected or not. The semantic expectancy effect was larger when the final word was sung on pitches forming an expected (tonic) rather than a less expected (subdominant) final chord. This result was taken to reflect attentional entrainment, as theorized in Jones’ dynamic attending theory [15,16]. According to this account, attentional fluctuations entrain to harmonic accents such as expected chords. This leads to an attentional peak when a tonic is heard. Greater attention at this point in time facilitates linguistic processing, hence the increased semantic priming effect. The same account has also been suggested to explain the music effect on visual processing [17] and phoneme monitoring [18]. Crucially, the nature of the stimulus is irrelevant in this account as long as it leads to attentional entrainment. This—again—is in sharp contrast to Patel’s [9] account which suggests that only syntactic manipulations in language will affect music processing.

### 1.4 The present paper: can language influence music perception? If so, how is music perception affected?

All the studies mentioned so far investigated musical influences on language or other tasks. We decided to reverse the direction of influence, i.e. we focus on linguistic influences on music processing. People listened to music while reading sentences or control arithmetic problems. The predictions of the syntax and the attention accounts outlined above are quite clear. Patel’s [9] SSIRH predicts that only syntactic manipulations will affect music harmony processing. Specifically, a challenging language syntax condition (ambiguous S-coordinations [19–21] in experiment 1 and object-relative clauses [2] in experiment 2) should tax musico-linguistic integration resources, leading to an impaired ability to harmonically integrate chords into an unfolding sequence. Non-syntactic processing challenges (difficult arithmetic operations in experiment 1 and semantic garden-path sentences [11] in experiment 2) should be without effect. The aforementioned attentional accounts, on the other hand, predict a non-specific effect, i.e. an effect for both the syntactic manipulations and the control manipulations.

Either way, the influence of a concurrent task on music perception could take two different forms since there are two aspects to harmony processing. First, the listener has to integrate chords in order to establish a harmonic key. Second, he/she must integrate chords with an already established key which is held online in some form of unification space (see §5.3 for details). We investigated these processes by presenting the challenging moment in concurrent tasks while the music piece modulated from an established harmonic key to a new key, i.e. when an old key has to be kept online without being reinforced by chords and a new key has to be established from the incoming chords. Chord sequences ended either on an authentic cadence typical for the first established key (probing whether the old key could be held online after it was no longer reinforced) or the second established key (probing whether a new key could be established after a critical moment in the concurrent task). The measure of interest which provides us a window into these processes is found in closure ratings. Participants are simply asked to rate their feeling of completeness (closure) regarding the music [22]. Closure is high if the last chords can be integrated into an available key. Reduced closure ratings indicate difficulty with harmonic integration—as expected in the case of syntactically challenging sentences compared to easier sentences.

## 2. Experiment 1 (exploratory): language syntax versus arithmetic difficulty

### 2.1 Method

#### 2.1.1 Participants

Fifty-eight participants were invited to take part in the experiment. One participant did not advance to the experiment as she did not understand the musical task after repeated chance-level practice trial performance. Three participants were rejected due to an error in counterbalancing.^2^ This leads to a final sample of 54 participants (19 men, 44 right handed). They were all Dutch native speakers, aged 23 on average (s.d.=6.3), with 3.9 years of musical training on average (s.d.=3.7). Thirty were self-described non-musicians, 19 amateur musicians, 5 semi-professional musicians. They were paid €16 or undergraduate course credits for their participation and were naive as to the purpose of the experiment.

#### 2.1.2 Design and material

**Figure 1.**
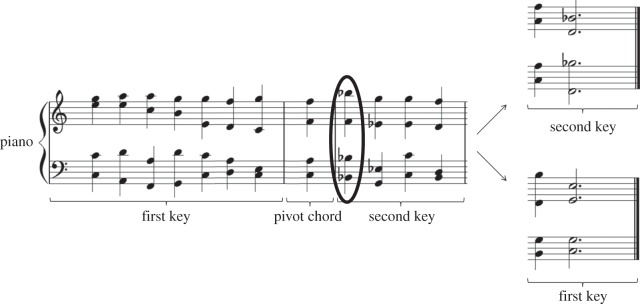
A sample music item in two versions. The top version ends in the second key (B-flat-major), the bottom version ends in the first key (C-major). The pivot chord (F-major chord) is part of both keys. The ninth chord (coinciding with the critical word in the concurrent language task and a manipulated operation in the concurrent arithmetic task) is encircled. From this chord onwards the first key has to be kept online without being reinforced by incoming chords (as tested by ratings of first key endings), and the second key has to be built up from new chords (as tested by ratings of second key endings). Bar lines denote boundaries between sections belonging to different harmonic keys.

We employed a 2 (Key: first key ending or second key ending) × 3 (Difficulty: ambiguous S-coordination, unambiguous S-coordination or NP-coordination) design for the musico-linguistic part of the experiment and a 2 (Key: first key ending or second key ending) × 2 (Difficulty: hard, easy) design for the musico-arithmetical part. All factors were manipulated within-subjects. All critical stimuli can be found in the appendices. Sound files are available as the electronic supplementary material. The task paired musical stimuli (harmonic sequences requiring a closure rating) with linguistic stimuli (visually presented sentences requiring a comprehension answer) or arithmetic stimuli (visually presented arithmetic formulae requiring a solution judgement). Auditory music stimuli were presented concurrently with visual language or arithmetic. The critical point in time was always the ninth position (highlighted or underlined in the examples below: figure 1, and examples 1 and 2), corresponding to the start of a new key in the musical material, the garden-path disambiguation in the ambiguous S-coordination sentences of the language stimuli and a difficult operation in the hard arithmetic trials.

##### 2.1.2.1 Music stimuli

The music stimuli consisted of chord sequences specifically composed for this experiment by the first author. As shown in figure 1, 10 items were composed beginning in C-major (seven chords), followed by a pivot chord which is part of both the C-major and B-flat-major keys (F-major chord or d-minor chord), followed by four chords in the B-flat-major key, ending with two chords forming an authentic cadence in C-major (first key ending) or B-flat-major (second key ending). These two versions of the 10 items were transposed twice (first key = D-major and second key = C-major; first key = B-flat major and second key = A-flat major). This resulted in 60 critical chord sequences (10 items × 2 endings × 3 transpositions). Next to the critical items, filler items in only one key (e.g. C-major) were constructed. For example, the second-key-region was transposed into C-major and a first-key-typical ending (C-major cadence) was chosen. Half of the filler sequences ended in an authentic cadence (requiring high closure ratings), half did not (dominant followed by supertonic or subdominant, requiring low closure ratings). This resulted in 60 filler chord sequences which allowed us to check whether participants paid attention to the musical task. Overall, participants were exposed to as many modulating (critical) sequences as non-modulating (filler) sequences. All chord sequences were played by a piano at a tempo of 96 bpm, consisting only of crotchets (quarter notes) except for the last chord which was made up of dotted minims (three-quarter notes).

##### 2.1.2.2 Language syntax stimuli

The critical language stimuli were a modified version of a stimulus set developed by Kerkhofs *et al.* [23]. As can be seen in example 1, 60 critical items were constructed in three versions each: an ambiguous S-coordination (garden-path version), an unambiguous S-coordination (intermediate difficulty version with a disambiguating comma before ‘**en**’, ***and***) and an NP-coordination (non-garden-path version). They were 14 words long. Pre-test results (see §2.1.3) revealed that the unambiguous S-coordination sentences were not perceived as significantly easier than the ambiguous S-coordination sentences. Therefore, we will concentrate on the contrast between the challenging syntax condition (ambiguous S-coordination) and the easier syntax condition (NP-coordination) here; see the electronic supplementary material for an analysis of critical trials involving unambiguous S-coordination sentences.

(1) Language example
(1a) ambiguous sentence-coordination (S-coordination, garden-path condition)
  $\text{De}|\text{chirurg}|\text{troostte}|\text{de}|\text{man}|\text{en}|\text{de}|\text{vrouw}|\underline{\text{legde}}|\text{haar}|\text{hand}|\text{op}|\text{zijn}|\text{voorhoofd.}$
  Translation: *The surgeon consoled the man* ***and*** *the woman* *put* *her hand on his forehead*.
(1b) noun-phrase-coordination (NP-coordination, non-garden-path condition)
  $\text{De}|\text{chirurg}|\text{troostte}|\text{de}|\text{man}|\text{en}|\text{de}|\text{vrouw}|\underline{\text{omdat}}|\text{de}|\text{operatie}|\text{niet}|\text{gelukt}|\text{was.}$
  Translation: *The surgeon consoled the man* ***and*** *the woman* *because* *the operation had not been successful*.

The beginning of each sentence (‘De chirurg troostte de man…’, *The surgeon consoled the man…*) was followed by ‘**en**’ (***and***) and a two-word noun phrase (‘de vrouw’, *the woman*), followed by a six-word ending of the sentence. This ending began either with a verb (ambiguous S-coordinations, ‘…legde haar hand op zijn voorhoofd.’, …*put* *her hand on his forehead.*) or without (NP-coordination, ‘…omdat de operatie niet gelukt was.’, …*because* *the operation had not been successful.*). Thus, these two stimulus versions only differed by the sentence ending.

The 60 filler items were also 14 words long and were made up of diverse syntactic constructions. 20 of the 60 filler stimuli included a NP-coordination, ensuring that overall participants read as many S-coordination sentences as NP-coordination sentences. Comprehension prompts of critical trials targeted the S- or NP-coordination ambiguity (e.g. 1: ‘De chirurg troostte alleen de man’. *The surgeon only consoled the man.*—true for S-coordination, false for NP-coordination). Filler comprehension prompts targeted various aspects of the filler sentence. Half the prompts required a ‘matches the sentence’ response, half required a ‘no match’ response.

##### 2.1.2.3 Arithmetic stimuli

The arithmetic stimuli were made up of seven numbers and seven operators each. Only additions and subtractions involving numbers equal to or below 10 were used after the first number. Interim solutions never exceeded 21 and were always positive integers. Arithmetic operations were classified as easy, hard or intermediate. Easy operations were additions or subtractions of the numbers 1, 2 or 10. Hard operations did not respect a 10-boundary, e.g. the addition of two numbers smaller than 10 whose sum is greater than 10. All other operations were seen as of intermediate difficulty. Note that the time pressure of rapid serial visual presentation meant that the arithmetic task was not too easy as shown by an accuracy of 84% on average.

There were 40 critical items, thus ensuring an equal number of arithmetic and linguistic critical trials in each Difficulty × Key-ending design cell (10 trials). As shown in example 2, their hard and easy versions were identical with regards to the first eight positions as well as the final two positions and the solution. The two stimulus versions differed according to the operations required at positions 9 (underlined in example 2) to 12. At the ninth position, in the hard version, the task required a difficult arithmetic operation, while in the easy version the operation was easy. In both hard and easy stimuli the operations after position 12 were easy. The 40 filler items were of the same length and only included easy and intermediate operations. Akin to comprehension prompts in the language task, prompts in the arithmetic task either matched the true solution (50% of trials) or differed from it by 1 (50% of trials).

(2) Arithmetic example
(2a) hard
  $\text{20 }|-|1|-|2|-|1|-|\underline{7}|-|2|+|1|=$
(2b) easy
  $\text{20 }|-|1|-|2|-|1|-|\underline{10}|+|1|+|1|=$

#### 2.1.3 Pre-test: the strength of the language syntax and arithmetic manipulations

A pre-test (*N*=24) showed that the difficulty manipulations in the language syntax task and the arithmetic task were both noticeable as revealed by trial difficulty ratings (Difficulty main effect; language: *p*<0.001; arithmetic: *p*<0.001; see the electronic supplementary material). As mentioned before, ambiguous and unambiguous S-coordination sentences were not perceived as significantly different [*t*_23_<1], suggesting the contrast ambiguous S-coordination versus NP-coordination will be more powerful in revealing linguistic influences on music processing. Therefore, we will focus on this contrast. Overall, the arithmetic manipulation was more salient (Manipulation × Difficulty interaction, *p*=0.028). This suggests that effects based on general task difficulty should be more easily visible in the arithmetic task. Thus, if there is an effect of language on music and it is due to general task difficulty, then the arithmetic control task should also show an effect.

#### 2.1.4 Procedure

**Figure 2.**
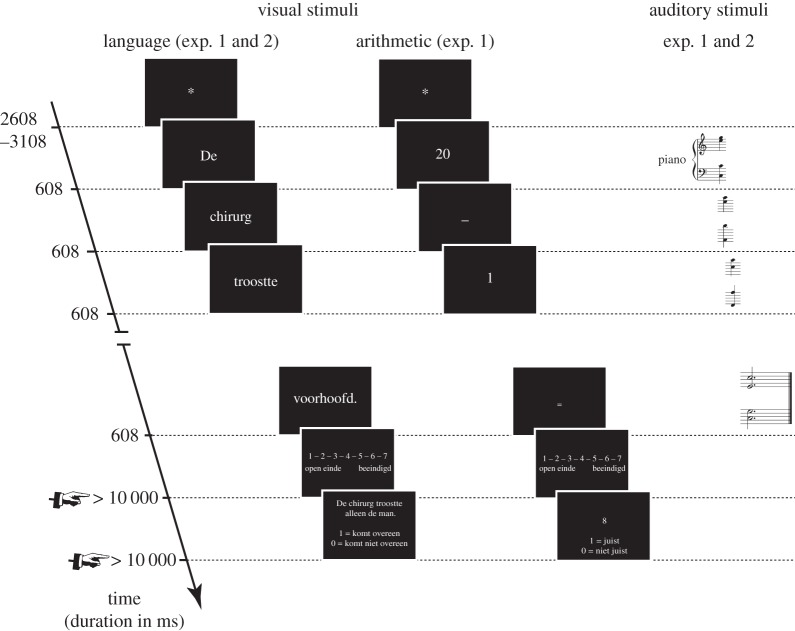
Stimulus sequence and timing in the visual and auditory modalities. On the left, a linguistic sequence (words from experiment 1) as used in experiments 1 and 2. In the middle, an arithmetic sequence as used in experiment 1. Note that comprehension prompts were presented only in one-third of the trials. On the right, an example of the chord sequences which were presented concurrently with the visual stimuli. Note that closure judgements on the chord sequence were part of each trial.

Participants were instructed to perform two tasks simultaneously: an auditory music task presented concurrently with a language task or an arithmetic task (figure 2). The type of concurrent task (language or arithmetic) was blocked and the order counterbalanced. An experimental session was organized as follows. Participants first completed an experimental block. Thereafter, they completed a musical background questionnaire and a working memory test (digit span [24]), followed by another experimental block. A testing session took approximately 2 h.

In the music task participants had to judge the closure, i.e. feeling of completeness, of a chord sequence on a 7-point Likert scale (1 = low closure, 7 = high closure). Closure judgements were required after each chord sequence. Participants were asked to answer a language/arithmetic comprehension prompt which followed one-third of the closure judgements (randomly chosen). Given the limited interest in the language/arithmetic task behaviour itself and in order to save time, we only included comprehension prompts on catch trials in order to ensure task adherence. Their unpredictable occurrence meant that the linguistic/arithmetic task had to be carried out throughout the experiment. Participants received feedback on their language/arithmetic accuracy every 20 trials.

In the musico-linguistic part of the experiment participants completed 120 trials, 40 of which were critical trials containing a garden-path (ambiguous S-coordination) or non-garden-path sentence (NP-coordination) in the language task and a harmonic sequence with modulation in the music task. Critical linguistic items were randomly paired with critical musical sequences.

Each language item was only shown in one version to each participant. The choice of item version was counterbalanced. Specifically, language item 1 might be presented in language condition A to participant 1, condition B to participant 2 and condition C to participant 3. For these three participants, the position of the item in the experiment was the same. Furthermore, the language item was paired with the same music stimulus for these three participants. For the next three participants, this item had a different position (determined by chance) and was matched with a different music stimulus (determined by chance). This way, a particular music–language item match could have no systematic influence on the results and the position in the experiment is counterbalanced for different language conditions. Each music item was presented once in every version to each participant. Trials were pseudo-randomized with the following constraints: (i) at least three trials between different versions of a music item, (ii) at most three filler or critical trials after each other, and (iii) at most three same answer conditions (prompt matching or not matching) after each other. The music stimuli were presented auditorily (608 ms per chord). The language stimuli were presented word by word (500 ms per word followed by 108 ms ISI) at the centre of the screen. The onset of a word presentation coincided with the onset of a chord.

In the musico-arithmetic part of the experiment participants completed 80 trials, 40 of which were critical trials containing a hard or an easy arithmetic problem in the arithmetic task and a harmonic sequence with modulation in the music task. The other half were filler trials. The combination of musical and arithmetic items, counterbalancing and trial randomization was done in the same way as described for the musico-linguistic task, except that only 80 musical sequences were used (original items and transposition down by two semitones, otherwise all versions of an item) because of the reduced trial count. The arithmetic stimuli were presented in a similar way as the language material (500 ms per number or operator followed by 108 ms ISI).

#### 2.1.5 Analysis

The closure ratings of critical trials were negatively skewed with a median rating of 6 (of 7), i.e. our data displayed a ceiling effect likely because all critical sequences ended on an authentic cadence. Therefore, an analysis based on mean ratings appeared inappropriate as the mean would not be a good representation of the *typical* closure rating behaviour. Instead, we based our analysis on the median. Specifically, we applied a subject-specific median-split analysis on ratings. For each participant, we coded ratings as either indicating high closure (subject-specific median of critical trial ratings or higher) or not. The analyses were performed on the proportion of trials in each condition which indicated high closure. This analysis strategy amplifies small differences in ratings between different concurrent task conditions while acknowledging that most critical trials are perceived as highly complete (high closure).

### 2.2 Results

#### 2.2.1 General task performance

Before presenting the crucial results of the critical trials, we first present evidence for good task adherence. Concerning the music task, using the filler trials which ended either in an authentic cadence (requiring high closure ratings) or not (requiring low closure ratings), we found that all participants had an equal or greater proportion of high-closure ratings for the authentic cadence endings than the no-cadence endings (*M*_authentic cadence_=0.81>*M*_no cadence_=0.12, difference s.d.=0.21). Therefore, all participants appear to have rated filler trial music as expected by music theory.

Language task accuracy (including filler and critical trials) was high in general (*M*=85%, s.d.=8%). As expected, the critical trial accuracy showed a clear difference between challenging ambiguous S-coordination (*M*=83%, s.d.=16%) and less challenging NP-coordination trials (*M*=90%, s.d.=15%; *t*_(53)_=2.28, *p*=0.027, _p_*η*^2^=0.089). Arithmetic task accuracy was similar in general (*M*=84%, s.d.=10%). As expected, the critical trial accuracy showed a clear difference between the hard (*M*=75%, s.d.=23%) and the easy arithmetic trials (*M*=92%, s.d.=10%; *t*_(53)_=5.25, *p*<0.001, _p_*η*^2^=0.342).

#### 2.2.2 Critical task performance

##### 2.2.2.1 Critical language results

**Figure 3.**
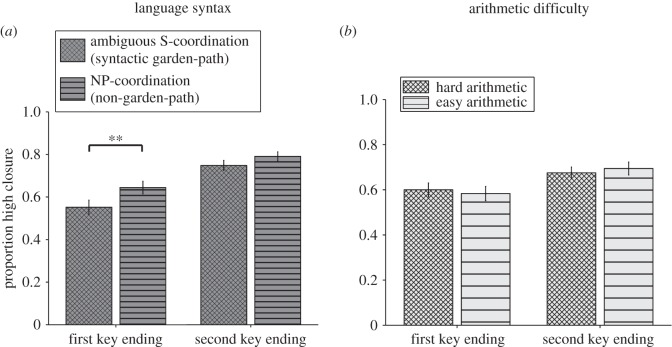
Experiment 1: closure ratings of critical trials. Participants were asked to rate their feeling of closure, i.e. completeness (*y*-axis), of harmonic sequences ending either in a way typical for a first established key or in a second-key-typical way (*x*-axis). Different bars represent concurrent task conditions. (*a*) In one block people solved the auditory music task while they were also asked to read sentences. We found an influence of language syntax on music harmony ratings. (*b*) In a different block, people solved the auditory task while they also performed an arithmetic task. The arithmetic manipulation was without effect. Significance levels represented as stars refer to simple main effect *t*-tests and do not imply significant interaction effects. Error bars=s.e.m. **p<0.01.

The critical closure data of the musico-linguistic part of the experiment were analysed in a 2 (Difficulty: ambiguous S-coordination, NP-coordination) × 2 (Key: first key ending, second key ending) within-subjects ANOVA; see figure 3*a*. There was a Difficulty main effect (*F*_1,53_=11.99, *p*=0.001, _p_*η*^2^=0.184). Otherwise, first key endings were less likely (*M*=0.60, s.d.=0.21) to receive a high closure rating than second-key endings (*M*=0.77, s.d.=0.14; Key, *F*_1,53_=51.74, *p*<0.001, _p_*η*^2^=0.494). These two factors did not interact (Key × Difficulty, *F*_1,53_=1.80, *p*=0.185, _p_*η*^2^=0.033). Still, as shown in figure 3*a*, the simple main effects revealed a Difficulty effect only for first key endings (*t*_53_=3.28, *p*=0.002, _p_*η*^2^=0.169), not for second key endings (*t*_53_=1.66, *p*=0.104, _p_*η*^2^=0.049).

##### 2.2.2.2 Critical arithmetic results

The critical closure data of the musico-arithmetic part of the experiment were analysed in a 2 (Difficulty: hard, easy) × 2 (Key: first key ending, second key ending) within-subjects ANOVA (figure 3*b*). There was no effect of arithmetical difficulty (*F*_1,53_<1). Otherwise, first key endings were less likely (*M*=0.59, s.d.=0.20) to receive a high closure rating than second-key endings (*M*=0.68, s.d.=0.18; Key, *F*_1,53_=13.69, *p*=0.001, _p_*η*^2^=0.205). These two factors did not interact (Key × Difficulty, *F*_1,53_<1).

##### 2.2.2.3 Language syntax versus arithmetic

In order to see whether the difficulty effect in the musico-linguistic task was significantly different from the one in the musico-arithmetic task we performed a three-way within-subjects ANOVA on the harmonic closure ratings with the factors Task (language, arithmetic), Difficulty (ambiguous S-coordination/hard, NP-coordination/easy) and Key (first key ending, second key ending). Indeed, the difficulty effect was greater in the language task than in the arithmetic task (Task × Difficulty, *F*_1,53_=5.20, *p*=0.027, _p_*η*^2^=0.089). Otherwise, all three factors showed a main effect (Task, *F*_1,53_=5.52, *p*=0.023, _p_*η*^2^=0.094) (Difficulty, *F*_1,53_=8.16, *p*=0.006, _p_*η*^2^=0.133) (Key, *F*_1,53_=41.33, *p*<0.001, _p_*η*^2^=0.438). The Key and Task factors interacted (*F*_1,53_=8.83, *p*=0.004, _p_*η*^2^=0.143) while Key and Difficulty did not (*F*_1,53_<1). The three-way interaction was marginally significant (Task × Difficulty × Key, *F*_1,53_=3.47, *p*=0.068, _p_*η*^2^=0.062).

### 2.3 Discussion: language syntax affects harmony judgements, arithmetic does not

The results of experiment 1 show that a syntactic garden-path effect can indeed influence harmony perception by reducing the ability to integrate chords, as shown by lower closure ratings at the end of a harmonic sequence. An arithmetic control condition, without syntactic demands but with a more salient difficulty manipulation, did not affect harmony perception. This shows that general trial difficulty is not involved in the syntax effect. Instead, as predicted by Patel’s [9] SSIRH, a syntactic challenge in language probably taxed shared musico-linguistic syntactic integration resources which in turn can then only sub-optimally process chords. A non-specific effect of linguistic or arithmetic difficulty on music ratings, as predicted by attentional accounts [11,15], was not observed. Note that the syntax effect was not specific to either type of music ending, suggesting that harmonic processing in general was affected, rather than holding a key online or establishing a new key.

The observed main effect of key is not of interest in this study. The fact that second-key endings more often receive high closure ratings than first-key endings likely reflects the relatively fast decline of key availability if a harmonic key is not reinforced by heard chords. Whether this is also the case without a concurrent task was investigated in a post-test which is part of the electronic supplementary material. Briefly, this post-test showed that no-cadence, first-key, and authentic cadence endings were rated very similarly without a concurrent language or arithmetic task. Second-key endings, however, less often received a high closure rating without a concurrent task compared to with, leading to a smaller difference between first-key and second-key ending ratings in this post-test. Still, the numerical trends were in the same direction, i.e. there is no indication in the post-test data that second-key endings are heard as less well closed compared to first-key endings. In sum, participants who only did the music task performed this task similarly to participants who did it while engaging in a second task at the same time.

However, a series of open questions remain concerning the effect of language on music ratings. Firstly, the difficulty in the language domain consisted in the syntactic re-analysis of a conjunction (‘**en**’, ***and***) held in memory. This sixth word either linked two sentences (S-coordination) or two noun-phrases (NP-coordination) depending on the ninth word in the sentence. The arithmetic control task required no such retrieval of an item from memory. Therefore, it could be hypothesized that the different effects of linguistic and arithmetic difficulty are based on a working memory mechanism: one either has to retrieve a temporally distant element or not. Re-analysing a word held in memory because of an unexpected new word could be thought to impair the ability to simultaneously hold chords in working memory. This in turn might impair the ability to use them for harmonic integration. In order to evaluate this alternative scenario we included digit span as a covariate in the analysis of the musico-linguistic task data. If working memory, rather than common syntactic integration resources, was responsible for the language effect, then the greater availability of working memory resources should lead to a reduction in the influence of language on music. An ANCOVA analysis with *z*-scored digit span as a measure of working memory did not support this scenario. Neither forward digit span, nor backward digit span, nor overall digit span significantly modulated any of the main effects or interactions (*ps*>0.1; see the electronic supplementary material). This supports a functional distinction between syntactic processing and general working memory [25–28].

A second open question relates to the specificity of the language manipulation. The linguistic contrast chosen in experiment 1 did not control for semantic or lexical differences after the eighth word of the sentence. Experiment 2 was designed to directly evaluate the influence of language syntax and language semantics while tightly controlling lexical items. We included a new syntactic contrast which matches lexical items between conditions (object- and subject-extracted relative clauses). The semantic manipulation consisted of semantic garden-path sentences (also called lexical garden-path sentences) in which a disambiguating word leads to the semantic re-interpretation of an ambiguous word held in memory. Note that semantic garden-path effects have previously been shown to be modulated by a music harmony manipulation [11]. This renders this particular semantic manipulation an ideal testing ground for the specificity of the syntactic effect encountered in experiment 1.

Finally, an additional reason for including a second experiment here is the *post hoc* nature of the analysis strategy we chose in experiment 1, i.e. the decision to focus on the proportion of high-closure ratings rather than raw ratings due to an unexpected ceiling effect. As opposed to the exploratory nature of experiment 1, experiment 2 was purely confirmatory and used the same analysis strategy.

## 3. Experiment 2 (confirmatory): language syntax versus language semantics

### 3.1 Method

#### 3.1.1 Participants

Sixty-two participants were invited to take part in the experiment (11 men, 54 right handed). None had participated in experiment 1. They were all Dutch native speakers, aged 21 on average (s.d.=3.0), with 3.3 years of musical training on average (s.d.=3.9). Forty-six were self-described non-musicians, 16 amateur musicians. They were paid € 12 or undergraduate course credits for their participation and were naive as to the purpose of the experiment.

#### 3.1.2 Design and material

We employed a 2 (Key: first key ending or second key ending) × 2 (Difficulty: object-relative clause, subject-relative clause) design for the language syntax part of the experiment and a 2 (Key: first key ending or second key ending) × 2 (Difficulty: semantic garden-path, non-garden-path) design for the semantic part of the experiment. All factors were manipulated within-subjects. As in experiment 1, auditory musical stimuli and visual linguistic stimuli were presented concurrently. The critical point in time was always the ninth position (underlined in examples 3 and 4 below), corresponding to the start of a new key in the musical material and the disambiguating word in the sentences.

##### 3.1.2.1 Music stimuli

We used the same music stimuli as in experiment 1.

##### 3.1.2.2 Language syntax stimuli

As can be seen in example 3, 40 critical items were constructed in two versions each: an object-extracted relative clause and a subject-extracted relative clause. Sentences were 14 words long and were identical apart from the relative clause verb (‘hielp’/‘hielpen’, ${\underline{\text{helped}}}_{\mathrm{singular}}/{\underline{\text{helped}}}_{\mathrm{plural}}$) which either agreed in number with the second noun-phrase (‘de zoon’, *the* *son*) in the object-extracted relative clause condition or the first noun phrase (‘de vrienden’, *the* *friends*) in the subject-extracted relative clause condition.

(3) Language example (syntactic manipulation)
(3a) object-extracted relative clause (OR)
  $\text{De}|\text{vrienden}|\text{die}|\text{de}|\text{zoon}|\text{op}|\text{de}|\text{been}|\underline{\text{hielp}}|\text{lieten}|\text{hem}|\text{het}|\text{gebouw}|\text{zien.}$
  Translation: *The friends* ***who*** *the son* *helped* *to get back on their feet let him see the building*.
(3b) subject-extracted relative clause (SR)
  $\text{De}|\text{vrienden}|\text{die}|\text{de}|\text{zoon}|\text{op}|\text{de}|\text{been}|\underline{\text{hielpen}}|\text{lieten}|\text{hem}|\text{het}|\text{gebouw}|\text{zien.}$
  Translation: *The friends* ***who*** *helped* *the son get back on his feet let him see the building*.

The 40 filler items included various syntactic constructions. Comprehension prompts of critical trials targeted the object- or subject-relative clause ambiguity (e.g. 3: ‘De vrienden hielpen de zoon’. *The friends helped the son*.—false for object-relative clause, true for subject-relative clause). Half the prompts required a ‘matches sentence’ response, half the opposite response.

##### 3.1.2.3 Language semantics stimuli

As can be seen in example 4, 40 critical items were constructed in two versions each: a semantic garden-path version and a non-garden-path version. Sentences were 14 words long and were identical apart from one manipulated word which occurred in positions two to seven (‘**muis**’/‘**veldmuis**’, ***mouse***/***field vole***) which was either ambiguous or not. The disambiguating word (‘rondlopen’, *run around*) was always the ninth word of the sentence.

(4) Language example (semantic manipulation)
(4a) semantic garden-path (GP)
  $\text{De}|\text{programmeur}|\text{liet}|\text{zijn}|\text{muis}|\text{op}|\text{de}|\text{tafel}|\underline{\text{rondlopen}}|\text{nadat}|\text{hij}|\text{hem}|\text{had}|\text{gevoerd.}$
  Translation: *The programmer let his* ***mouse*** *run around* *on the table after he had fed it*.
(4b) non-garden-path (non-GP)
  $\text{De}|\text{programmeur}|\text{liet}|\text{zijn}|\text{veldmuis}|\text{op}|\text{de}|\text{tafel}|\underline{\text{rondlopen}}|\text{nadat}|\text{hij}|\text{hem}|\text{had}|\text{gevoerd.}$
  Translation: *The programmer let his* ***field vole*** *run around* *on the table after he had fed it*.

Additionally, 40 filler items were included. They used various syntactic constructions and avoided semantic ambiguities. Comprehension prompts of critical trials targeted various parts of the sentence (e.g. 4: ‘De muis was gevoerd’. *The mouse had been fed.*—true). Half the prompts matched the sentence.

#### 3.1.3 Pre-test 1: sentence completions show the intended misinterpretation of semantic material

Before starting the main experiment we conducted two pre-tests with two purposes. Firstly, we wanted to choose the 40 best semantic items out of an original set of 60. Items were included if they exhibited the typical semantic garden-path pattern of a semantically ambiguous word being initially misinterpreted until a disambiguating word changes the interpretation. Below we only report the analysis involving the 40 items which were used in experiment 2. Secondly, as in experiment 1, we wanted to establish the strength of the manipulations in the syntactic and the semantic parts of the experiment.

In a first pre-test (*N*=54) participants were asked to complete sentence beginnings which did not include a disambiguating word (words 1 to 8, e.g. ‘De programmeur liet zijn **muis** op de tafel…’, *The programmer let his* ***mouse***…). Sentence completions revealed that the intended word interpretation was overwhelmingly adopted in the non-garden-path condition (93.6% of trials) while this hardly happened in the semantic garden-path condition (13.3%). This is exactly the pattern expected of semantic garden-path sentences (see the electronic supplementary material).

#### 3.1.4 Pre-test 2: the strength of the syntactic and semantic manipulations

The aim of the second pre-test was to establish the strength of the difficulty manipulations of the syntactic and the semantic items. Participants (*N*=24) read sentences word by word while the timing of word presentation was under their control (self-paced reading). Afterwards they rated trials for difficulty. The semantic manipulation led to higher reading times from the disambiguating word onwards in the semantic garden-path condition (compared to non-garden-path condition). The difference was 44 ms (*p*=0.005) for the critical disambiguating word and 85 ms (*p*<0.001) for the post-critical word. Overall, this resulted in a greater perceived difficulty with these sentences (*p*=0.003). Moreover, the syntactic manipulation was equally salient as seen in reading times (reading time difference on critical ninth word: 64 ms (*p*=0.009), on post-critical word: 58 ms (*p*=0.008)) and difficulty ratings (*p*=0.001; see the electronic supplementary material). Thus, if we observe differences between the syntactic and the semantic manipulations these cannot be attributed to quantitatively different processing requirements of the two manipulations as they were matched in this respect.

#### 3.1.5 Procedure

The task was the same as in experiment 1. The type of manipulation (syntax or semantics) was blocked and the order counterbalanced. Experimental sessions did not include a working memory test. A testing session took approximately 90 min.

#### 3.1.6 Analysis

The analysis was the same as in experiment 1.

### 3.2 Results

#### 3.2.1 General task performance

Before presenting the crucial critical trial data, we first show evidence of good task-adherence. Concerning the music task, using the filler trials we found, as expected, that all participants had a greater proportion of high-closure ratings for the authentic cadence endings than the no-cadence endings (*M*_authentic cadence_=0.76>*M*_no cadence_=0.09, difference s.d.=0.18).

Accuracy during the syntax part was high in general (*M*=70%, s.d.=9%). As expected, prompts after object-relative clauses were answered less accurately (*M*=37%, s.d.=24%) than those after subject-relative clauses (*M*=76%, s.d.=19%; *t*_61_=9.80, *p*<0.001, _p_*η*^2^=0.661). Accuracy during the semantics part was higher in general (*M*=84%, s.d.=7%). There was no significant accuracy difference between garden-path (*M*=86%, s.d.=14%) and non-garden-path trials (*M*=85%, s.d.=13%; *t*_61_<1). However, this null effect should not be taken to mean that the semantic garden-path effect was somehow absent. Instead, it reflects the fact that the comprehension prompts in the semantics part often did not target the semantic ambiguity of the sentences. As pre-test 2 showed, using the same comprehension prompts, critical semantics trials still exhibited a garden-path effect both in reading time and difficulty ratings, showing that it is not necessary to focus comprehension prompts on the garden-path manipulation in order for it to have an effect.

#### 3.2.2 Critical task performance

##### 3.2.2.1 Critical results of the syntax part

**Figure 4.**
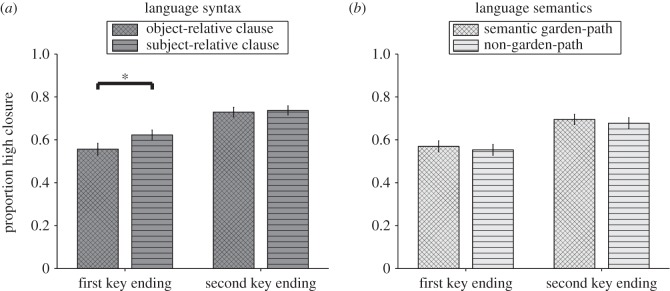
Experiment 2: closure ratings of critical trials. Participants were asked to rate their feeling of closure (*y*-axis) of first-key-typical endings or second-key-typical endings (*x*-axis). While people solved the auditory music task they were also asked to read sentences. (*a*) We found an influence of a syntactic manipulation on music harmony ratings. (*b*) A semantic garden-path manipulation was without effect. Significance levels represented as stars refer to simple main effect *t*-tests and do not imply significant interaction effects. Error bars=s.e.m. *p<0.05.

The critical closure data of the syntax part were analysed in a 2 (Difficulty: object-relative clause, subject-relative clause) × 2 (Key: first key ending, second key ending) within-subjects ANOVA (figure 4*a*). Object-relative clauses led to significantly fewer (*M*=0.64, s.d.=0.15) high closure ratings than subject-relative clauses (*M*=0.68, s.d.=0.14; Difficulty, *F*_1,61_=5.45, *p*=0.023, _p_*η*^2^=0.082). Otherwise, first key endings were less likely (*M*=0.59, s.d.=0.17) to receive a high closure rating than second-key endings (*M*=0.73, s.d.=0.15; Key, *F*_1,61_=38.53, *p*<0.001, _p_*η*^2^=0.387). These two factors did not interact (Key × Difficulty, *F*_1,61_=2.48, *p*=0.121, _p_*η*^2^=0.039). Still, as shown in figure 4*a*, the simple main effects revealed a Difficulty effect only for first key endings (*t*_61_=2.51, *p*=0.015, _p_*η*^2^=0.094), not for second key endings (*t*_61_<1).

##### 3.2.2.2 Critical results of the semantics part

The critical closure data of the semantics part were analysed in a 2 (Difficulty: semantic garden-path, non-garden-path) × 2 (Key: first key ending, second key ending) within-subjects ANOVA (figure 4*b*). Closure ratings did not differ between semantic garden-path (*M*=0.63, s.d.=0.16) and non-garden-path trials (*M*=0.62, s.d.=0.15; *F*_1,61_<1). Otherwise, first-key endings were less likely (*M*=0.56, s.d.=0.16) to receive a high closure rating than second-key endings (*M*=0.69, s.d.=0.15; Key, *F*_1,61_=27.00, *p*<0.001, _p_*η*^2^=0.307). These two factors did not interact (Key × Difficulty, *F*_1,61_<1).

##### 3.2.2.3 Language syntax versus language semantics

In order to see whether the influence of the syntax manipulation on music ratings was significantly different from the influence of the semantics manipulation we performed a three-way within-subjects ANOVA with the factors Manipulation (syntax, semantics), Difficulty (object-relative clause/semantic garden-path, subject-relative clause/non-garden-path) and Key (first key ending, second key ending). Indeed, the difficulty effect was different in the syntax part and the semantics part (Manipulation × Difficulty, *F*_1,61_=4.10, *p*=0.047, _p_*η*^2^=0.063). Otherwise, only one main effect was significant (Key, *F*_1,61_=44.56, *p*<0.001, _p_*η*^2^=0.422) (Manipulation, *F*_1,61_=3.85, *p*=0.054, _p_*η*^2^=0.059) (Difficulty, *F*_1,61_<1) (Manipulation × Key, *F*_1,61_<1) (Difficulty × Key, *F*_1,61_=1.39, *p*=0.242, _p_*η*^2^=0.022) (Manipulation × Difficulty × Key, *F*_1,61_<1).

### 3.3 Discussion: language syntax affects harmony judgements, semantics does not

In line with the exploratory first experiment, experiment 2 has shown that a syntactic challenge in language can influence harmony perception. Moreover, it was shown that a non-syntactic language manipulation, namely a semantic garden-path manipulation, has no such effect. This pattern of results is indicative of a shared syntactic resource account [9], not a general attention mechanism [11,15]. Interestingly, our findings suggest that semantic processing itself does not influence music harmony perception in contrast to the influence of music itself on semantic processing found by [11]. Potential reasons for this will be discussed.

The difference between the syntactic effect and the semantic effect is remarkable. These two manipulations were rated as equally salient and led to very similar reading time changes. Nonetheless, the nature of the manipulation had different effects on the concurrent music perception. As discussed in §2.3, one could also propose a memory-based explanation for the difference between syntactic and semantic effects on music. Differences in the distance between the ambiguous word and the disambiguating ninth word could be responsible. However, when looking at these distances it becomes clear that the semantic manipulation (distance ambiguous–disambiguating word: *M*=4.08, s.d.=1.65, 95% CI based on bootstrapping with 50 000 sample=[3.58;4.60]) was intermediate between the two syntactic manipulations (experiment 1: always three words between ‘**en**’, ***and***, and the ninth word; experiment 2: distance between ‘**die**’, ***who*** and disambiguating ninth word, *M*=4.98 words, s.d.=1.03, 95% CI=[4.65;5.28]). This makes a memory based explanation for the difference between the semantic and the syntactic effects unlikely.

Having investigated what kind of manipulations do exert an influence on music harmony perception, we will now turn towards a more detailed analysis of the syntax effect itself. So far, we were unable to distinguish an effect on either of two harmonic sub-processes: holding a key online (reflected in first-key ending ratings) and establishing a new key (reflected in second-key ending ratings). The greater statistical power afforded by combining the data of the syntactic manipulations of both experiments might illuminate this issue.

## 4. Experiments 1 and 2: combined analysis of the syntax effect

### 4.1 Results

**Figure 5.**
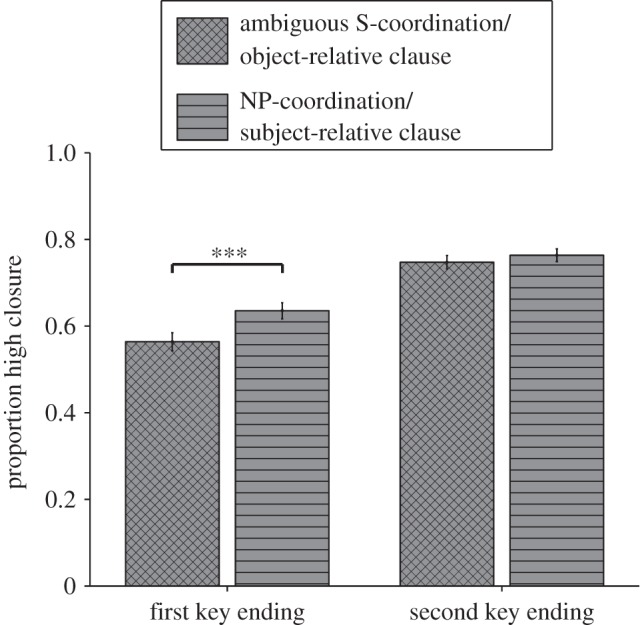
Experiments 1 and 2: closure ratings of critical trials. Participants were asked to rate their feeling of closure (*y*-axis) of first-key-typical endings or second-key-typical endings (*x*-axis). While people solved the auditory music task they were also asked to read sentences. The language syntax effect was specific to harmonic sequences ending in a first-key-typical way. Error bars=s.e.m. ***p<0.001.

We combined the data of the syntax manipulations of experiment 1 (ambiguous S-coordination, NP-coordination) and experiment 2 (object-relative clause, subject-relative clause) to form a single Difficulty factor (figure 5). A 2 (Experiment: 1, 2) × 2 (Difficulty) × 2 (Key: first key ending, second key ending) mixed between- and within-subjects ANOVA exhibited the main effects of Difficulty and Key which we observed before (Difficulty, *F*_1,114_=17.63, *p*<0.001, _p_*η*^2^=0.134) (Key, *F*_1,114_=89.47, *p*<0.001, _p_*η*^2^=0.440). The factor Experiment was without effect (Experiment, *F*_1,114_<1) (Difficulty × Experiment, *F*_1,114_=1.50, *p*=0.224, _p_*η*^2^=0.013) (Key × Experiment, *F*_1,114_<1) (Difficulty × Key × Experiment, *F*_1,114_<1). However, the greater power revealed a previously non-significant interaction between Difficulty and Key (*F*_1,114_=4.21, *p*=0.042, _p_*η*^2^=0.036). Follow-up *t*-tests showed that the difficulty effect was specific to first-key-typical endings (*t*_115_=4.09, *p*<0.001, _p_*η*^2^=0.127) while it was non-significant for second-key-typical endings (*t*_115_=1.43, *p*=0.155, _p_*η*^2^=0.018). Note that including musical training as a covariate does not change the pattern of these results. In the overall ANCOVA *z*-scored musical training years did not modulate any of the main effects or interactions (*ps*>0.2); see the electronic supplementary material. In the electronic supplementary material, we also provide analyses by items.

### 4.2 Discussion: a first-key-specific syntax effect

Using the greater power afforded by the combination of the data of experiments 1 and 2, we have shown that the syntax effect is specific to harmonic sequences ending in the first key. This suggests that during music listening shared music–language resources are involved in holding a key online in order to interpret incoming chords in its context. Building up a new key seems to be processed by different resources. We will discuss the implications of these findings in the general discussion. One should bear in mind that experiment 1 was exploratory in nature, and thus the result of the combined analysis should be replicated.

The comparison also showed that the syntax manipulation in experiment 1 appears to be numerically stronger (_p_*η*^2^=0.169 for the difficulty effect related to first key endings) than in experiment 2 (_p_*η*^2^=0.094). Looking at the comprehension accuracy data reveals that this small difference might be related to participants sometimes giving up a full syntactic analysis of the very challenging object-relative clauses (accuracy=37%) but do so less in the challenging ambiguous S-coordination trials (accuracy=83%). This might indicate that syntactic integration resources can also be taxed too much, leading to incomplete parsing of the sentence [29]. The details of such nonlinear effects should be worked out in future studies. For the present purposes we can conclude that challenging syntax processing, even when it leads to incomplete syntactic interpretations, draws resources away from the harmonic integration of chords.

## 5. General discussion

### 5.1 Summary

We present two experiments which investigated whether music harmony processing can be influenced by a concurrent language or arithmetic task. Patel’s [9] SSIRH hypothesizes that previously observed musical influences on the processing of language syntax are due to shared musico-linguistic resources involved in syntactic integration. Therefore, under this account music harmony should be influenced only if the concurrent task involves a syntactic manipulation. Our results support this prediction. While two different language syntax contrasts (ambiguous S-coordination versus NP-coordination and object-relative clause versus subject-relative clause) modulated harmonic closure ratings, arithmetical difficulty as well as a semantic garden-path manipulation had no such effect. This contradicts accounts relating previous music–language interactions solely to general attention mechanisms which would have predicted an influence of the arithmetic and semantic control manipulations.

The functional role of shared syntax resources in terms of harmonic processing was further investigated by looking at the syntax effect on ratings of different kinds of musical endings. This analysis revealed that a syntactic challenge in language reduces the music listener’s ability to maintain an already established key online for the purpose of harmonic integration of chords. Building up a new key was unaffected. In what follows, we will discuss these results.

### 5.2 Novel support for shared syntactic integration resources

The present findings offer novel support for Patel’s [9] SSIRH. The novelty stems from the use of a musical measure of interest, as opposed to previously used linguistic measures such as language comprehension accuracy [2], reading times [5] or word judgement times [3]. Our findings suggest that the direction of influence is not crucial for interference effects. Music can influence language and vice versa. This is in line with studies which measured brain activity during music and language processing in similar paradigms. For example, in an electroencephalogram (EEG) event-related potential (ERP) study, Steinbeis & Koelsch [6] found language processing in the form of the left anterior negativity to be altered by a concurrent harmonic violation (see also [4]). They also found the early right anterior negativity elicited by an unexpected chord to be influenced by language syntax errors (but see [4]). This was taken as evidence for a change in musical processing as a result of a linguistic syntactic violation. Clearly, both offline measures as used here or by Fedorenko *et al.* [2] and online measures of processing [3–6,8] reveal a mutual influence between music and language.

However, there is also a negative deflection in the EEG signal which peaks around 650 ms after stimulus onset and which is elicited by an unexpected chord. This deflection is often called the N5 and it can be modulated by a language semantics manipulation [6], suggesting an effect of language semantics on music harmony in contradiction to our results in experiment 2. However, the N5 is not elicited in some experiments [4] or participant groups [30]. Moreover, other EEG studies working with similar interference paradigms failed to find an interaction between music harmonics and language semantics [4,31] (see also [32]). Therefore, it appears difficult to interpret the apparent contradiction between Steinbeis & Koelsch’s [6] ERP result and our semantics null effect in experiment 2. More research is needed in order to better characterize the N5. Until then we are inclined to rely on the straightforward interpretation of our behavioural measure to indicate an absence of semantic influences on harmonic processing. Only language syntax appears to reliably influence music harmony behaviour.

### 5.3 The role of shared resources in the music network

In the current experiment only the first harmonic key which was held online was affected by a concurrent language syntax manipulation. This could be taken to reflect shared resources’ role as a specifically *syntactic* working memory in the brain’s music network [33]. In terms of language, this aspect of working memory makes ‘syntactic information actively available over sustained periods of the sentence while new information is being processed continuously’ [26], p. 88. An additional role of syntactic working memory could be to hold a *harmonic* *key* online while new chords are integrated. This process might be impaired if concurrently processing complex sentences restricts the syntactic working memory resources left available for music.

Alternatively, music and language might share syntactic unification resources [34], p. 416/417. According to this account, lexical items are retrieved from memory into a ‘unification workspace’ in which ‘constituent structures spanning the whole utterance are formed’ by combining items according to syntactic principles, i.e. by unifying them. The more demanding unification operation associated with disambiguating words of garden-path sentences might impair the ability of shared resources to keep a harmonic key in the unification workspace. Therefore, the key is no longer a potential site with which incoming chords can be unified. This study cannot distinguish between these two accounts.

### 5.4 Non-syntactic overlap between music and language?

We found no evidence for a general attentional mechanism driving the linguistic influence on music, as both control conditions were without effect. Thus, the nature of the manipulation (syntactic rather than something else) appears important. However, according to Perruchet & Poulin-Charronat’s [11] attentional load account influences from one domain on the other only emerge if enough attentional resources are left to process both. In the present experiments, it could be argued that critical language syntax trials might be overall less demanding than control task trials, leaving more attention to music perception. However, there is no evidence for such an account here. As the difficulty ratings of the pre-tests revealed, critical language syntax trials were sometimes seen as *overall* easier (experiment 1) or harder (experiment 2) than their control task counterparts (*ps*≤0.001; see electronic supplementary material). Thus, in this study, there is no linear relationship between the effect of a task on music perception and its overall difficulty. General attention as well as shared syntactic resources might be involved in musical influences on language [5,11], while language influencing music relies solely on syntactic resources. This proposal should be tested in the future.

Next to attention based explanations, an error based explanation of previous studies’ results [3,4,6] was put forward by Rogalsky *et al.* [35]. However, our study only used well-formed sentences and musical sequences without errors, as done in previous behavioural studies investigating musical influences on language [2,5]. Similarly, our results could also be said to be due to a working memory based mechanism (not to be confused with the notion of syntactic working memory [26,33]). In order to hold a key online listeners might need working memory resources which the syntactic/semantic reinterpretation of a previously encountered word taxes. However, phonological working memory was not associated with the syntax effect found in experiment 1, and working memory effects would lead to similar semantic and syntactic garden-path influences on harmonic processing. Instead, we found that only the latter affects music ratings. This leads us to favour the SSIRH put forward by Patel [9] as the likely mechanism behind our results, rather than attentional accounts [11,15,16], an error processing explanation [35], or a working memory account.

## 6. Conclusion

This study found evidence that music and language share resources for syntactic integration [9]. Our results suggest that challenging language syntax impacts on the ability to integrate chords into an already established key. It appears that music and language are processed in a similar way by the human brain due to a key commonality: they are both structured sequences, i.e. their constituent elements relate in a rule-governed way to each other. Shared syntax resources are responsive to structured sequences in general—whether they are linguistic or musical in nature.

## Supplementary Material

Kunert, Willems, & Hagoort, accepted_RSOS_supplementary material_v.4.0.docx Detailed analyses of Experiment 1's pretest, Experiment 1's analysis including the intermediate difficulty condition, Experiment 2's pre-tests, Experiment 1's ANCOVA analysis with working memory as a co-variate, combined analysis of Experiment 1's language data and Experiment 2's syntax data with musical training as a co-variate, and by-items analyses, post-test analysis (without concurrent task next to music task), additional order effect analysis of main experiments.

## Supplementary Material

dataAll_Exp1_Arithmetic.csv The data acquired in experiment 1's arithmetic part.

## Supplementary Material

dataAll_Exp1_Language.csv The data acquired in experiment 1's language part.

## Supplementary Material

dataAll_Exp2_Semantics.csv The data acquired in experiment 2's semantics part.

## Supplementary Material

dataAll_Exp2_Syntax.csv The data acquired in experiment 2's syntax part.

## Supplementary Material

Exp1_Analysis.m The matlab analysis code for experiment 1 which produces Figure 3.

## Supplementary Material

Exp2_Analysis.m The matlab analysis code for experiment 2 which produces Figure 4.

## Supplementary Material

Exp1and2_Analysis.m The matlab analysis code for the language syntax conditions in experiments 1 and 2 which produces Figure 5.

## Supplementary Material

audio_files_v2.zip

## Footnotes

1 In the Western tonal tradition, music harmony refers to the organization of pitches in terms of scales and chords (simultaneous soundings of several pitches). There are twelve pitches per octave, adjacent pitches being separated by one semitone. Pitches occupying the same ordinal position in different octaves are said to belong to the same pitch class (e.g. all the C-notes on a piano). Each scale (e.g. C-major scale) consists of seven pitch classes (in-key notes, e.g. C, D, E, F, G, A, B). When playing in a musical key (e.g. C-major key) these seven pitch classes are emphasized and the five remaining pitch classes (out-of-key notes, e.g. C#, D#, F#, G#, A#) are relatively unexpected; the same applies to the chords based on these tones.

2 Including these three participants does not alter the findings of the study. Notably, the crucial Difficulty main effect in the musico-linguistic part of the experiment was still significant (*F*_1,56_=12.59, *p*=0.001, _p_*η*^2^=0.184), as was the Task × Difficulty interaction when including Task as a factor (*F*_1,56_=5.50, *p*=0.023, _p_*η*^2^=0.089).

## Data accessibility

The datasets supporting this article have been uploaded as part of the electronic supplementary material. Matlab code for reproducing figures 3, 4, and 5 is also provided as part of the electronic supplementary material.

## Appendix A

**A.1. Experiments 1 and 2: music**

Only the C-major/B-flat major version is shown. Two transpositions were made of these items. See §2.1.2.1. for details. no. item 1 2

**Table 1.** Experiment 1: language syntax.

no.	ambiguous S-coordination	unambiguous S-coordination	NP-coordination
1	De chirurg troostte de man en de vrouw legde haar hand op zijn voorhoofd.	De chirurg troostte de man, en de vrouw legde haar hand op zijn voorhoofd.	De chirurg troostte de man en de vrouw omdat de operatie niet gelukt was.
2	De voorzitter bedankte de sponsor en de trainer bestelde lachend een biertje voor iedereen.	De voorzitter bedankte de sponsor, en de trainer bestelde lachend een biertje voor iedereen.	De voorzitter bedankte de sponsor en de trainer zonder de goede spelers te noemen.
3	De mannequin kuste de ontwerper en de fotograaf pakte een champagne en wat kaviaar.	De mannequin kuste de ontwerper, en de fotograaf pakte een champagne en wat kaviaar.	De mannequin kuste de ontwerper en de fotograaf hoewel zij hen niet leuk vond.
4	De rector ondervroeg de leraar en de leerling volgde het gesprek vanaf de gang.	De rector ondervroeg de leraar, en de leerling volgde het gesprek vanaf de gang.	De rector ondervroeg de leraar en de leerling over het ongeval op het schoolplein.
5	De gevangene gijzelde de priester en de bewaker riep geschrokken om hulp te krijgen.	De gevangene gijzelde de priester, en de bewaker riep geschrokken om hulp te krijgen.	De gevangene gijzelde de priester en de bewaker in de omsingelde gevangenis van Breda.
6	De weduwe bedankte de organist en de predikant bekeek de mensen die waren gekomen.	De weduwe bedankte de organist, en de predikant bekeek de mensen die waren gekomen.	De weduwe bedankte de organist en de predikant voor de geschikte dienst op Allerzielen.
7	De bedrijfsleider kalmeerde de gast en de ober bracht het bord naar de keuken.	De bedrijfsleider kalmeerde de gast, en de ober bracht het bord naar de keuken.	De bedrijfsleider kalmeerde de gast en de ober nadat het licht ineens weer aanging.
8	De redacteur prees de fotograaf en de journalist bekeek bewonderend de foto’s van vluchtelingen.	De redacteur prees de fotograaf, en de journalist bekeek bewonderend de foto’s van vluchtelingen.	De redacteur prees de fotograaf en de journalist voor het artikel over de vluchtelingen.
9	De sheriff beschermde de boer en de knecht verdedigde de boerderij tegen Jonsons bende.	De sheriff beschermde de boer, en de knecht verdedigde de boerderij tegen Jonsons bende.	De sheriff beschermde de boer en de knecht tegen de aanval van Johnson’s bende.
10	De grimeur schminkte de schrijver en de interviewer besprak de vragen die zouden komen.	De grimeur schminkte de schrijver, en de interviewer besprak de vragen die zouden komen.	De grimeur schminkte de schrijver en de interviewer voordat de camera’s begonnen te filmen.
11	De verdachte beledigde de rechter en de advocaat belde ontstemd het kantoor zonder succes.	De verdachte beledigde de rechter, en de advocaat belde ontstemd het kantoor zonder succes.	De verdachte beledigde de rechter en de advocaat in de rechtszaal voor het publiek.
12	De eigenaar prees de kok en de ober floot zachtjes een heel bekend liedje.	De eigenaar prees de kok, en de ober floot zachtjes een heel bekend liedje.	De eigenaar prees de kok en de ober op het jubileumfeest van zijn restaurant.
13	De dirigent bekritiseerde de cellist en de pianist smeet zijn partituur op de grond.	De dirigent bekritiseerde de cellist, en de pianist smeet zijn partituur op de grond.	De dirigent bekritiseerde de cellist en de pianist omdat zij niet harmonieus samen speelden.
14	De portier bespioneerde de chef en de secretaresse belde heimelijk vrienden bij de politie.	De portier bespioneerde de chef, en de secretaresse belde heimelijk vrienden bij de politie.	De portier bespioneerde de chef en de secretaresse op order van de rijke baron.
15	De dief beschoot de juwelier en de agent riskeerde zijn leven door te intervenieren.	De dief beschoot de juwelier, en de agent riskeerde zijn leven door te intervenieren.	De dief beschoot de juwelier en de agent met een gestolen vuurwapen uit Amerika.
16	De regisseur bespotte de nieuwslezer en de weerman vervloekte de opzet van het programma.	De regisseur bespotte de nieuwslezer, en de weerman vervloekte de opzet van het programma.	De regisseur bespotte de nieuwslezer en de weerman nadat hij veel wijn had gedronken.
17	De winnares omhelsde de sponsor en de trainer groette het publiek op de tribune.	De winnares omhelsde de sponsor, en de trainer groette het publiek op de tribune.	De winnares omhelsde de sponsor en de trainer met een glimlach op haar lippen.
18	De rechter berispte de verdachte en de advocaat bedacht snel redenen voor een verdaging.	De rechter berispte de verdachte, en de advocaat bedacht snel redenen voor een verdaging.	De rechter berispte de verdachte en de advocaat omdat zij onafgebroken met elkaar praatten.
19	De presentator introduceerde de schrijver en de criticus maakte grijnzend buigingen naar het publiek.	De presentator introduceerde de schrijver, en de criticus maakte grijnzend buigingen naar het publiek.	De presentator introduceerde de schrijver en de criticus aan het publiek in de zaal.
20	De stalker achtervolgde de danseres en de manager opende vlug de deur van metaal.	De stalker achtervolgde de danseres, en de manager opende vlug de deur van metaal.	De stalker achtervolgde de danseres en de manager hoewel hij eigenlijk naar huis moest.
21	De politieman ondervroeg de koerier en de infiltrant achterhaalde later zonder problemen zijn naam.	De politieman ondervroeg de koerier, en de infiltrant achterhaalde later zonder problemen zijn naam.	De politieman ondervroeg de koerier en de infiltrant in een onderzoek naar internationaal terrorisme.
22	De gravin wenkte de koetsier en de lakei droeg zuchtend de koffers naar huis.	De gravin wenkte de koetsier, en de lakei droeg zuchtend de koffers naar huis.	De gravin wenkte de koetsier en de lakei vanuit de koets die geparkeerd stond.
23	De presentator omarmde de zanger en de zangeres zong huilend hun trieste eerste hit.	De presentator omarmde de zanger, en de zangeres zong huilend hun trieste eerste hit.	De presentator omarmde de zanger en de zangeres terwijl hun trieste eerste hit weerklonk.
24	De hulpverlener informeerde de arts en de brandweerman bracht gehaast het slachtoffer buiten gevaar.	De hulpverlener informeerde de arts, en de brandweerman bracht gehaast het slachtoffer buiten gevaar.	De hulpverlener informeerde de arts en de brandweerman over de gevaren van witte asbest.
25	De tovenaar bewaakte de koningin en de prinses haalde het toverboek om te helpen.	De tovenaar bewaakte de koningin, en de prinses haalde het toverboek om te helpen.	De tovenaar bewaakte de koningin en de prinses met zijn toverstafje voor de draak.
26	De voorbijganger bevrijdde het kind en de vrouw schreeuwde de longen uit haar lijf.	De voorbijganger bevrijdde het kind, en de vrouw schreeuwde de longen uit haar lijf.	De voorbijganger bevrijdde het kind en de vrouw uit de auto die gekanteld was.
27	De boswachter berispte de padvinder en de hopman doofde gauw het vuurtje met zand.	De boswachter berispte de padvinder, en de hopman doofde gauw het vuurtje met zand.	De boswachter berispte de padvinder en de hopman nadat hij hun vuur had gezien.
28	De toerist fotografeerde de visser en de reisleider vertelde een verhaal over de visserij.	De toerist fotografeerde de visser, en de reisleider vertelde een verhaal over de visserij.	De toerist fotografeerde de visser en de reisleider zonder hun om toestemming te vragen.
29	De dichter bezong de zwerver en de dronkaard prees luidkeels de schoonheid van Amsterdam.	De dichter bezong de zwerver, en de dronkaard prees luidkeels de schoonheid van Amsterdam.	De dichter bezong de zwerver en de dronkaard met een melodie uit zijn kinderjaren.
30	De professor belde de aannemer en de architect eiste direct een onderzoek naar woningcorporaties.	De professor belde de aannemer, en de architect eiste direct een onderzoek naar woningcorporaties.	De professor belde de aannemer en de architect om over de villa te praten.
31	De klant bedankte de bedrijfsleider en de bediende vroeg de kassabon voor de trui.	De klant bedankte de bedrijfsleider, en de bediende vroeg de kassabon voor de trui.	De klant bedankte de bedrijfsleider en de bediende voor de ruil van haar trui.
32	De lerares begroette de leerling en de moeder beschreef uitvoerig de thuissituatie zonder vader.	De lerares begroette de leerling, en de moeder beschreef uitvoerig de thuissituatie zonder vader.	De lerares begroette de leerling en de moeder in het klaslokaal van haar klas.
33	De pastoor zegende de stuurman en de kapitein bedankte de geestelijke voor zijn zorgen.	De pastoor zegende de stuurman, en de kapitein bedankte de geestelijke voor zijn zorgen.	De pastoor zegende de stuurman en de kapitein voordat het schip in zee voer.
34	De chauffeur vervoerde de baron en de butler bracht de bagage naar het kasteel.	De chauffeur vervoerde de baron, en de butler bracht de bagage naar het kasteel.	De chauffeur vervoerde de baron en de butler na de bruiloft van de prins.
35	De actrice vervloekte de stuntman en de producent gooide woedend zijn dikke sigaar weg.	De actrice vervloekte de stuntman, en de producent gooide woedend zijn dikke sigaar weg.	De actrice vervloekte de stuntman en de producent toen zij op haar kamer zat.
36	De burgemeester ondervroeg de leraar en de onderzoeker onderkende de voordelen van het onderwijsplan.	De burgemeester ondervroeg de leraar, en de onderzoeker onderkende de voordelen van het onderwijsplan.	De burgemeester ondervroeg de leraar en de onderzoeker over de ontwikkeling van jonge schoolkinderen.
37	Het Kamerlid bespotte de interviewer en de minister herhaalde minachtend de vragen van journalisten.	Het Kamerlid bespotte de interviewer, en de minister herhaalde minachtend de vragen van journalisten.	Het Kamerlid bespotte de interviewer en de minister net nadat het interview uitgezonden was.
38	De lijfwacht beschermde de president en de generaal beval direct de omgeving te onderzoeken.	De lijfwacht beschermde de president, en de generaal beval direct de omgeving te onderzoeken.	De lijfwacht beschermde de president en de generaal tegen de bedreiging door de demonstranten.
39	De automobilist raakte de voetganger en de fietser viel geschrokken op de natte straat.	De automobilist raakte de voetganger, en de fietser viel geschrokken op de natte straat.	De automobilist raakte de voetganger en de fietser met zijn onachtzaam geopende deur aan.
40	De tuinman bespiedde het dienstmeisje en de butler pakte verrekijkers om mee te doen.	De tuinman bespiedde het dienstmeisje, en de butler pakte verrekijkers om mee te doen.	De tuinman bespiedde het dienstmeisje en de butler met een verrekijker vanuit de tuin.
41	De clown ontvluchtte de goochelaar en de acrobaat beklom de ladder naar de trapeze.	De clown ontvluchtte de goochelaar, en de acrobaat beklom de ladder naar de trapeze.	De clown ontvluchtte de goochelaar en de acrobaat terwijl het hele publiek hard lachte.
42	De suppoost waarschuwde de student en de studente stopte snel drugs in haar jas.	De suppoost waarschuwde de student, en de studente stopte snel drugs in haar jas.	De suppoost waarschuwde de student en de studente voor de drugssmokkelaar die gezocht werd.
43	De psychiater observeerde de patient en de assistent schreef zorgvuldig de medische gegevens op.	De psychiater observeerde de patient, en de assistent schreef zorgvuldig de medische gegevens op.	De psychiater observeerde de patient en de assistent met bewakingscamera’s in de grote behandelkamer.
44	De huisvrouw zoende de kennis en het kind bekeek nieuwsgierig de mensen die voorbijliepen.	De huisvrouw zoende de kennis, en het kind bekeek nieuwsgierig de mensen die voorbijliepen.	De huisvrouw zoende de kennis en het kind howel zij hen niet goed kende.
45	De directeur ontsloeg de werknemer en de afdelingsbaas riskeerde zijn baan door het ontslag.	De directeur ontsloeg de werknemer, en de afdelingsbaas riskeerde zijn baan door het ontslag.	De directeur ontsloeg de werknemer en de afdelingsbaas omdat de hele afdeling gesloten werd.
46	De burgemeester loofde de wethouder en de ondernemer liet meteen een fles cognac bezorgen.	De burgemeester loofde de wethouder, en de ondernemer liet meteen een fles cognac bezorgen.	De burgemeester loofde de wethouder en de ondernemer voor hun inzet voor het weeshuis.
47	De koningin beloonde de lakei en de hofdame kreeg onmiddellijk een kleur van opwinding.	De koningin beloonde de lakei, en de hofdame kreeg onmiddellijk een kleur van opwinding.	De koningin beloonde de lakei en de hofdame allebei met twee zware gouden daalders.
48	De reiziger vervloekte de piloot en de stewardess verzorgde keurig een passagier met hoofdpijn.	De reiziger vervloekte de piloot, en de stewardess verzorgde keurig een passagier met hoofdpijn.	De reiziger vervloekte de piloot en de stewardess na de noodlanding in de zee.
49	De astronaut groette de technicus en de monteur opende behoedzaam de sluis naar binnen.	De astronaut groette de technicus, en de monteur opende behoedzaam de sluis naar binnen.	De astronaut groette de technicus en de monteur voordat hij in de capsule klom.
50	De fan belaagde de drummer en de gitarist riep ontzet naar de ontbrekende beveiliging.	De fan belaagde de drummer, en de gitarist riep ontzet naar de ontbrekende beveiliging.	De fan belaagde de drummer en de gitarist met een mes in de hand.
51	De commissaris bedreigde de parkeerwacht en de rechercheur vertrok waarbij hij de deur dichtsmeet.	De commissaris bedreigde de parkeerwacht, en de rechercheur vertrok waarbij hij de deur dichtsmeet.	De commissaris bedreigde de parkeerwacht en de rechercheur met een aangifte voor illegaal gokken.
52	De archeoloog betaalde de indiaan en de graver stopte alle spullen in een koffer.	De archeoloog betaalde de indiaan, en de graver stopte alle spullen in een koffer.	De archeoloog betaalde de indiaan en de graver uit zijn eigen fonds zonder steun.
53	De dichter belaagde de criticus en de redacteur besloot meteen een rectificatie te plaatsen.	De dichter belaagde de criticus, en de redacteur besloot meteen een rectificatie te plaatsen.	De dichter belaagde de criticus en de redacteur nadat de negatieve kritiek was gepubliceerd.
54	De verpleger verschoonde de junk en de zwerfster waste mopperend haar gezicht met zeep.	De verpleger verschoonde de junk, en de zwerfster waste mopperend haar gezicht met zeep.	De verpleger verschoonde de junk en de zwerfster in het tehuis voor arme mensen.
55	De kapelaan vermaande de koorknaap en het hulpje wist nauwelijks zijn lachen te bedwingen.	De kapelaan vermaande de koorknaap, en het hulpje wist nauwelijks zijn lachen te bedwingen.	De kapelaan vermaande de koorknaap en het hulpje omdat zij ‘s nachts veel kletsten.
56	De medicijnman besprenkelde de bezetene en het opperhoofd goot voorzichtig olie over het masker.	De medicijnman besprenkelde de bezetene, en het opperhoofd goot voorzichtig olie over het masker.	De medicijnman besprenkelde de bezetene en het opperhoofd met sacraal water uit de bron.
57	De priester offerde de slavin en de slaaf bewierookte dromerig het mysterieuze stenen beeld.	De priester offerde de slavin, en de slaaf bewierookte dromerig het mysterieuze stenen beeld.	De priester offerde de slavin en de slaaf aan zijn goden die zoiets vorderden.
58	De volgeling vereerde de goeroe en de ingewijde luisterde ademloos naar zijn gepassioneerde toespraak.	De volgeling vereerde de goeroe, en de ingewijde luisterde ademloos naar zijn gepassioneerde toespraak.	De volgeling vereerde de goeroe en de ingewijde zonder hun boek te hebben gelezen.
59	De activist besmeurde de lijfwacht en de officier morste koffie op zijn smetteloze uniform.	De activist besmeurde de lijfwacht, en de officier morste koffie op zijn smetteloze uniform.	De activist besmeurde de lijfwacht en de officier met veel melk van zijn boerderij.
60	De fakir betoverde de toeschouwer en de danseres vertoonde geamuseerd haar tropische sensuele buikdans.	De fakir betoverde de toeschouwer, en de danseres vertoonde geamuseerd haar tropische sensuele buikdans.	De fakir betoverde de toeschouwer en de danseres nadat de olifant eindelijk gekalmeerd was.

**Table 2.** Experiment 1: arithmetic.

no.	easy	hard
1	1+1+2−1+10+2−1=	1+1+2−1+9+1+1=
2	20−1−2−1−10+1+1=	20−1−2−1−7−2+1=
3	9+1−1−1+2+10−2=	9+1−1−1+6+2+2=
4	7+2+1+1−1−2−2=	7+2+1+1−5+2−2=
5	10+10−1−1+2−1−1=	10+10−1−1+3−2−1=
6	4−1+1+2+2+2+2=	4−1+1+2+7+1−2=
7	2+2−1+2+1+1+1=	2+2−1+2+6−2−1=
8	18−2−1−1−10+2+1=	18−2−1−1−6−2+1=
9	6+1+2−2+1+1+2=	6+1+2−2+5+1−2=
10	13−2+1+1−1−2−2=	13−2+1+1−8+1+2=
11	1+1+2+1+1+1+2=	1+1+2+1+8−2−2=
12	19−2−1−1−2−2−1=	19−2−1−1−7+1+1=
13	20−1−10−2+1+1+2=	20−1−10−2+8−2−2=
14	7+1+2+2−2−2−2=	7+1+2+2−5+1−2=
15	10+1−2−1+2+1+1=	10+1−2−1+5−2+1=
16	9+1+1+1−10+1+2=	9+1+1+1−7−2+2=
17	11−1−2−2+2+2+1=	11−1−2−2+8−2−1=
18	8+2+2+1−10+2+2=	8+2+2+1−7−1+2=
19	18−10−2−1+1+2+1=	18−10−2−1+7−2−1=
20	6−2−2+10−2−2−2=	6−2−2+10−7−1+2=
21	12−1−10+2+2−2−1=	12−1−10+2+8−10+1=
22	5+10+1+1−1−2−2=	5+10+1+1−9+2+2=
23	3+1+1+1+1+2+1=	3+1+1+1+7−2−1=
24	1+1+2+10−1−2−2=	1+1+2+10−8+1+2=
25	4+1+1+2+1+2+2=	4+1+1+2+6+1−2=
26	7+10+1−2−1−2−1=	7+10+1−2−7+2+1=
27	2+1+1+1+2+2+2=	2+1+1+1+7+1−2=
28	8+1+2+2−1−2−2=	8+1+2+2−6−1+2=
29	10+10−2−10+2+2+2=	10+10−2−10+5−1+2=
30	6−1−1+10−1−2−1=	6−1−1+10−6+1+1=
31	2+2+2+1+2+1+2=	2+2+2+1+6+1−2=
32	7+1+2+2−10+2+2=	7+1+2+2−7−1+2=
33	10+2+2−10+1+1+2=	10+2+2−10+8−2−2=
34	8−1−1+10−2−1−1=	8−1−1+10−7+2+1=
35	1+10−1−2+1+1+2=	1+10−1−2+5+1−2=
36	3+1+10+2−2−1+1=	3+1+10+2−9+10−1=
37	9+1−2−2+1+1+1=	9+1−2−2+5−1−1=
38	4+1+1+10−1−2−1=	4+1+1+10−7+2+1=
39	10−1−1−2+2+1+1=	10−1−1−2+7−2−1=
40	3+1+2+10−1−10+1=	3+1+2+10−8−1−1=

**Table 3.** Experiment 2: language syntax.

no.	object-relative clause	subject-relative clause
1	Hij weet dat de tantes die de jongen begroette op een eiland zijn geweest.	Hij weet dat de tantes die de jongen begroetten op een eiland zijn geweest.
2	De heel aantrekkelijke vrouwen die de ervaren soldaat omhelsde waren erg geliefd bij iedereen.	De heel aantrekkelijke vrouwen die de ervaren soldaat omhelsden waren erg geliefd bij iedereen.
3	De oude generaals die de kolonel met opzet misleidde genoten bekendheid in de media.	De oude generaals die de kolonel met opzet misleidden genoten bekendheid in de media.
4	De profs die de naieve tegenstander zonder schaamte uitlachte begingen toen een grote vergissing.	De profs die de naieve tegenstander zonder schaamte uitlachten begingen toen een grote vergissing.
5	De vrienden die de zoon op de been hielp lieten hem het gebouw zien.	De vrienden die de zoon op de been hielpen lieten hem het gebouw zien.
6	De twee broers die de iets oudere zuster bijstond stonden bekend om hun succes.	De twee broers die de iets oudere zuster bijstonden stonden bekend om hun succes.
7	Twan hoorde dat de inbrekers die de bandiet doodde zeiden berouwloos te hebben gehandeld.	Twan hoorde dat de inbrekers die de bandiet doodden zeiden berouwloos te hebben gehandeld.
8	De rijke blanken die de eenzame zwarte ongevraagd uitnodigde betaalden voor al het eten.	De rijke blanken die de eenzame zwarte ongevraagd uitnodigden betaalden voor al het eten.
9	De goed betaalde advocaten die de rechter blind vertrouwde namen het meest verstandige besluit.	De goed betaalde advocaten die de rechter blind vertrouwden namen het meest verstandige besluit.
10	De officieren die de luitenant met weinig succes geruststelde werden meegenomen naar het politiebureau.	De officieren die de luitenant met weinig succes geruststelden werden meegenomen naar het politiebureau.
11	De heiligen die de wijze drie jaar lang bewonderde spraken vol lof over hem.	De heiligen die de wijze drie jaar lang bewonderden spraken vol lof over hem.
12	De bazen die de nieuwe werknemer uiteindelijk toch bezocht gingen reizen maken door Europa.	De bazen die de nieuwe werknemer uiteindelijk toch bezochten gingen reizen maken door Europa.
13	De boze bewoners die de agent uit Breda verbaasde schreeuwden kwaad naar de voorbijgangers.	De boze bewoners die de agent uit Breda verbaasden schreeuwden kwaad naar de voorbijgangers.
14	De juffrouwen die de student met veel passie kuste maakten zich uit de voeten.	De juffrouwen die de student met veel passie kusten maakten zich uit de voeten.
15	De meisjes die de zeer oude grootvader graag knuffelde leken zich goed te vermaken.	De meisjes die de zeer oude grootvader graag knuffelden leken zich goed te vermaken.
16	De geestelijken die de huisarts in een restaurant ontving vroegen of alles goed was.	De geestelijken die de huisarts in een restaurant ontvingen vroegen of alles goed was.
17	De jonge meneren uit Amsterdam die de vrouw verleidde kregen ruzie met de politieagent.	De jonge meneren uit Amsterdam die de vrouw verleidden kregen ruzie met de politieagent.
18	De gevaarlijke dieren die de hongerige wilde stiekem besloop kwamen uit een donker bos.	De gevaarlijke dieren die de hongerige wilde stiekem beslopen kwamen uit een donker bos.
19	De dronken gasten die de net getrouwde echtgenoot vermaakte zongen mee met de muziek.	De dronken gasten die de net getrouwde echtgenoot vermaakten zongen mee met de muziek.
20	De artsen die de client zonder veel moeite begreep raadden hem een operatie aan.	De artsen die de client zonder veel moeite begrepen raadden hem een operatie aan.
21	De schattige baby die de ouders bijna niet herkenden groette hen met een glimlach.	De schattige baby die de ouders bijna niet herkende groette hen met een glimlach.
22	De leerling die de leraren en leraressen herhaaldelijk opbelden was lang niet meer gezien.	De leerling die de leraren en leraressen herhaaldelijk opbelde was lang niet meer gezien.
23	De minister die de politici met onverwachte onthullingen choqueerden besloot zijn nevenfunctie niet neer te leggen.	De minister die de politici met onverwachte onthullingen choqueerde besloot zijn nevenfunctie niet neer te leggen.
24	De onbekende vreemde die de omstreden pastoors meermaals benaderden volgde hun en praatte beleefd.	De onbekende vreemde die de omstreden pastoors meermaals benaderde volgde hun en praatte beleefd.
25	De machtige priester die de bisschoppen in Rome toespraken werkte gedreven aan een boek.	De machtige priester die de bisschoppen in Rome toesprak werkte gedreven aan een boek.
26	De man die de directeuren met een brief feliciteerden droeg een nieuw zwart pak.	De man die de directeuren met een brief feliciteerde droeg een nieuw zwart pak.
27	De vriendelijke dame die de kunstenaars van harte bedankten klonk erg onder de indruk.	De vriendelijke dame die de kunstenaars van harte bedankte klonk erg onder de indruk.
28	De vreemde psycholoog die de patientes zonder onderbreking aanstaarden droeg een bril uit Italie.	De vreemde psycholoog die de patientes zonder onderbreking aanstaarde droeg een bril uit Italie.
29	De dichter die de nog onbekende schrijvers publiekelijk aanmoedigden wilde zij beter leren kennen.	De dichter die de nog onbekende schrijvers publiekelijk aanmoedigde wilde zij beter leren kennen.
30	De opscheppende deskundige uit Canada die de Amerikanen corrigeerden wilde de resultaten meteen presenteren.	De opscheppende deskundige uit Canada die de Amerikanen corrigeerde wilde de resultaten meteen presenteren.
31	De uitgeputte chef die de vele klanten kennelijk negeerden maakte een totaal chagrijnige indruk.	De uitgeputte chef die de vele klanten kennelijk negeerde maakte een totaal chagrijnige indruk.
32	De ambtenaar die de kennissen op de straat ontweken leek moe en vooral gestrest.	De ambtenaar die de kennissen op de straat ontweek leek moe en vooral gestrest.
33	De paranoide patiente die de bezoekers uit Groningen uithoorden verkeerde nog in kritieke toestand.	De paranoide patiente die de bezoekers uit Groningen uithoorde verkeerde nog in kritieke toestand.
34	De stoere vent die de jongeren zonder reden aanvielen sloeg snel op de vlucht.	De stoere vent die de jongeren zonder reden aanviel sloeg snel op de vlucht.
35	Journalisten beweerden dat de deelnemer die de medewerkers geloofden niet alle details kon weten.	Journalisten beweerden dat de deelnemer die de medewerkers geloofde niet alle details kon weten.
36	De blonde prins die de grote zwarte paarden vreesden droeg alleen zeer kleurrijke kleding.	De blonde prins die de grote zwarte paarden vreesde droeg alleen zeer kleurrijke kleding.
37	De hoestende jongeman die de dokters in opleiding zagen was eigenlijk helemaal niet ziek.	De hoestende jongeman die de dokters in opleiding zag was eigenlijk helemaal niet ziek.
38	De eigenaar die de arbeiders op het werk stoorden moest zich naar buiten haasten.	De eigenaar die de arbeiders op het werk stoorde moest zich naar buiten haasten.
39	Niemand voorzag dat de dochter die de spelers verbluften graag een nieuwe taal leerde.	Niemand voorzag dat de dochter die de spelers verblufte graag een nieuwe taal leerde.
40	De erg bezige verpleegster die de verwarde zieken zochten had afspraken met de chirurgen.	De erg bezige verpleegster die de verwarde zieken zocht had afspraken met de chirurgen.

**Table 4.** Experiment 2: language semantics.

no.	semantic garden-path	non-garden-path
1	De kokkin dacht aan het gerecht dat haar aanklaagde voor een zeer ernstig delict.	De kokkin dacht aan het gerechtshof dat haar aanklaagde voor een zeer ernstig delict.
2	De alcoholist had last van zijn kater die miauwde en daar niet mee ophield.	De alcoholist had last van zijn kat die miauwde en daar niet mee ophield.
3	De pianospeler keek naar de toets om hem in te vullen maar hij wist geen antwoorden.	De pianospeler keek naar de Cito-toets om hem in te vullen maar hij wist geen antwoorden.
4	De programmeur liet zijn muis op de tafel rondlopen nadat hij hem had gevoerd.	De programmeur liet zijn veldmuis op de tafel rondlopen nadat hij hem had gevoerd.
5	De generaal vond dat een kwartier niet genoeg plek was voor de groep soldaten.	De generaal vond dat een kamer niet genoeg plek was voor de groep soldaten.
6	De oude prof hield een bal met veel bezoekers, muziek en ook veel eten.	De oude prof hield een feest met veel bezoekers, muziek en ook veel eten.
7	De musicus kende de noten die hij had gegeten omdat hij ook bioloog was.	De musicus kende de kokosnoten die hij had gegeten omdat hij ook bioloog was.
8	De grimmige douanier eiste de tol en ander speelgoed om het te laten checken.	De grimmige douanier eiste de teddybeer en ander speelgoed om het te laten checken.
9	De veer in de vleugel van een grote burcht werd door een schoonmaakster weggeveegd.	De veer in de verdieping van een grote burcht werd door een schoonmaakster weggeveegd.
10	Door verrekijkers konden de jachtopzieners de manen van Jupiter erg duidelijk uit elkaar houden.	Door verrekijkers konden de jachtopzieners de satellieten van Jupiter erg duidelijk uit elkaar houden.
11	Niemand kon vermoeden dat de palm zo erg bloedde dat de hand eraf moest.	Niemand kon vermoeden dat de handpalm zo erg bloedde dat de hand eraf moest.
12	De werklozen zochten een goede baan om te schaatsen en gezellig bier te drinken.	De werklozen zochten een goede ijsbaan om te schaatsen en gezellig bier te drinken.
13	De industrieel wilde een kanaal maken om meer toeschouwers met zijn reclame te bereiken.	De industrieel wilde een tv-kanaal maken om meer toeschouwers met zijn reclame te bereiken.
14	De visser gebruikte een aas om het lange kaartspel te winnen en te beeindigen.	De visser gebruikte een klaverenaas om het lange kaartspel te winnen en te beeindigen.
15	De spin hoorde eigenlijk naar buiten op haar bagagedrager waarop ze vaak boodschappen plaatste.	De snelbinder hoorde eigenlijk naar buiten op haar bagagedrager waarop ze vaak boodschappen plaatste.
16	De bioloog zag het blad, begon het te lezen en vergat zijn dure experiment.	De bioloog zag het weekblad, begon het te lezen en vergat zijn dure experiment.
17	In Hollywood kon je veel sterren in de hemel zien toen de straatverlichting uitviel.	In Hollywood kon je veel sterrenbeelden in de hemel zien toen de straatverlichting uitviel.
18	De kampeerder had veel kolen nodig voor de salade omdat zijn vrouw wilde meeeten.	De kampeerder had veel groenten nodig voor de salade omdat zijn vrouw wilde meeeten.
19	De arts vermoedde dat het kaakje niet goed smaakte want zijn kinderen haatten het.	De arts vermoedde dat het koekje niet goed smaakte want zijn kinderen haatten het.
20	Het vak dat hij moest geven was een erfstuk en deel van een wandkast.	Het schap dat hij moest geven was een erfstuk en deel van een wandkast.
21	De politicus kon de grond voor zijn grote vergissing niet goed aan iedereen uitleggen.	De politicus kon de reden voor zijn grote vergissing niet goed aan iedereen uitleggen.
22	Het rooster was al lang klaar maar de barbecuegasten waren nog steeds niet gearriveerd.	Het grillrooster was al lang klaar maar de barbecuegasten waren nog steeds niet gearriveerd.
23	De cowboy nam de poot van zijn nieuwe bureau en zaagde een stukje eraf.	De cowboy nam de tafelpoot van zijn nieuwe bureau en zaagde een stukje eraf.
24	De automonteur voelde de stroom die rond vijftig volt sterk was en hem blesseerde.	De automonteur voelde de elektriciteit die rond vijftig volt sterk was en hem blesseerde.
25	De dierenoppasser vond de lange slang tussen ander tuingereedschap en begon met het afspuiten.	De dierenoppasser vond de lange tuinslang tussen ander tuingereedschap en begon met het afspuiten.
26	De restaurantbezoeker ging naar het buffet dat van hout was en er duur uitzag.	De restaurantbezoeker ging naar het kastje dat van hout was en er duur uitzag.
27	Het vertrek van zijn overleden moeder was nog kleiner en donkerder dan het zijne.	Het kamertje van zijn overleden moeder was nog kleiner en donkerder dan het zijne.
28	De toneelspeler had een rol nodig voor het deeg nadat vorige taarten waren mislukt.	De toneelspeler had een plank nodig voor het deeg nadat vorige taarten waren mislukt.
29	De beurs die de studente kreeg was een designproduct en was in Italie gemaakt.	De portemonnee die de studente kreeg was een designproduct en was in Italie gemaakt.
30	De matroos keek naar de maten van zijn T-shirts voordat hij op reis ging.	De matroos keek naar de kleuren van zijn T-shirts voordat hij op reis ging.
31	De rijdster koos per ongeluk de vaart die onderstroomde en ging meteen over kop.	De rijdster koos per ongeluk de waterweg die onderstroomde en ging meteen over kop.
32	De wetenschapper had een nieuwe vlam die niet uitblust ontwikkeld en presenteerde hem overal.	De wetenschapper had een nieuw vuur dat niet uitblust ontwikkeld en presenteerde het overal.
33	Het wereldberoemde koor kon helaas niet meer goed gerestaureerd worden zonder miljoenen te investeren.	De wereldberoemde kloostergang kon helaas niet meer goed gerestaureerd worden zonder miljoenen te investeren.
34	De net geboren koe die de boer had gelaten stond op de grote bergweide.	De net geboren koe die de scheet had gelaten stond op de grote bergweide.
35	De zin van zijn leven die hij had gedicht maakte hem in Nederland bekend.	De uitspraak van zijn leven die hij had gedicht maakte hem in Nederland bekend.
36	De jonge patient wilde de pil niet meteen lezen omdat hij over ziektes ging.	De jonge patient wilde de tekst niet meteen lezen omdat hij over ziektes ging.
37	De band die de fietsenmaker met zijn zus voelde werd niet door haar beantwoord.	De bloedband die de fietsenmaker met zijn zus voelde werd niet door haar beantwoord.
38	De politieman zag de aanslag die op zijn tanden zat na het koffie drinken.	De politieman zag de kleur die op zijn tanden zat na het koffie drinken.
39	De Turk constateerde dat de sirene niet meer leefde en Odysseus weer vrij was.	De Turk constateerde dat de toverheks niet meer leefde en Odysseus weer vrij was.
40	Het koetje dat pasgeboren was kon bijna al vliegen hoewel zijn veren kort waren.	Het vogeltje dat pasgeboren was kon bijna al vliegen hoewel zijn veren kort waren.

## References

[1] Brown S, Jordania J 2013 Universals in the world’s musics. *Psychol. Music* 41, 229–248. (doi:10.1177/0305735611425896)

[2] Fedorenko E, Patel A, Casasanto D, Winawer J, Gibson E 2009 Structural integration in language and music: evidence for a shared system. *Mem. Cogn.* 37, 1–9. (doi:10.3758/MC.37.1.1) 10.3758/MC.37.1.1 19103970

[3] Hoch L, Poulin-Charronnat B, Tillmann B 2011 The influence of task-irrelevant music on language processing: syntactic and semantic structures. *Front. Psychol.* 2, 112 (doi:10.3389/fpsyg.2011.00112) 21713122 10.3389/fpsyg.2011.00112 PMC3112335

[4] Koelsch S, Gunter TC, Wittfoth M, Sammler D 2005 Interaction between syntax processing in language and in music: an ERP study. *J. Cogn. Neurosci.* 17, 1565–1577. (doi:10.1162/089892905774597290) 16269097 10.1162/089892905774597290

[5] Slevc LR, Rosenberg JC, Patel AD 2009 Making psycholinguistics musical: self-paced reading time evidence for shared processing of linguistic and musical syntax. *Psychon. Bull. Rev.* 16, 374–381. (doi:10.3758/16.2.374) 19293110 10.3758/16.2.374 PMC2658747

[6] Steinbeis N, Koelsch S 2008 Shared neural resources between music and language indicate semantic processing of musical tension-resolution patterns. *Cereb. Cortex* 18, 1169–1178. (doi:10.1093/cercor/bhm149) 17720685 10.1093/cercor/bhm149

[7] Kunert R, Slevc LR 2015 A commentary on: ‘Neural overlap in processing music and speech’. *Front. Hum. Neurosci.* 9, 330 (doi:10.3389/fnhum.2015.00330) 26089792 10.3389/fnhum.2015.00330 PMC4452821

[8] Kunert R, Willems RM, Casasanto D, Patel AD, Hagoort P 2015 Music and language syntax interact in Broca’s area: an fMRI study. *PLoS ONE* 10, e0141069 (doi:10.1371/journal.pone.0141069) 26536026 10.1371/journal.pone.0141069 PMC4633113

[9] Patel AD 2008 Music, language, and the brain. Oxford, UK: Oxford University Press.

[10] Poulin-Charronnat B, Bigand E, Madurell F, Peereman R 2005 Musical structure modulates semantic priming in vocal music. *Cognition* 94, B67–B78. (doi:10.1016/j.cognition.2004.05.003) 15617668 10.1016/j.cognition.2004.05.003

[11] Perruchet P, Poulin-Charronnat B 2013 Challenging prior evidence for a shared syntactic processor for language and music. *Psychon. Bull. Rev.* 20, 310–317. (doi:10.3758/s13423-012-0344-5) 23180417 10.3758/s13423-012-0344-5

[12] Peretz I, Coltheart M 2003 Modularity of music processing. *Nat. Neurosci.* 6, 688–691. (doi:10.1038/nn1083) 12830160 10.1038/nn1083

[13] Koelsch S, Gunter TC, von Cramon DY, Zysset S, Lohmann G, Friederici AD 2002 Bach speaks: a cortical ‘language-network’ serves the processing of music. *Neuroimage* 17, 956–966. (doi:10.1006/nimg.2002.1154) 12377169

[14] Patel AD, Gibson E, Ratner J, Besson M, Holcomb PJ 1998 Processing syntactic relations in language and music: an event-related potential study. *J. Cogn. Neurosci.* 10, 717–733. (doi:10.1162/089892998563121) 9831740 10.1162/089892998563121

[15] Jones M, Boltz M 1989 Dynamic attending and responses to time. *Psychol. Rev.* 96, 459–491. (doi:10.1037/0033-295X.96.3.459) 2756068 10.1037/0033-295x.96.3.459

[16] Large EW, Jones MR 1999 The dynamics of attending: how people track time-varying events. *Psychol. Rev.* 106, 119–159. (doi:10.1037/0033-295X.106.1.119)

[17] Escoffier N, Tillmann B 2008 The tonal function of a task-irrelevant chord modulates speed of visual processing. *Cognition* 107, 1070–1083. (doi:10.1016/j.cognition.2007.10.007) 18076873 10.1016/j.cognition.2007.10.007

[18] Bigand E, Tillmann B, Poulin B, D’Adamo DA, Madurell F 2001 The effect of harmonic context on phoneme monitoring in vocal music. *Cognition* 81, B11–B20. (doi:10.1016/S0010-0277(01)00117-2) 11525485 10.1016/s0010-0277(01)00117-2

[19] Frazier L 1987 Syntactic processing: evidence from Dutch. *Nat. Lang. Linguist. Theory* 5, 519–559. (doi:10.1007/BF00138988)

[20] Hoeks JCJ, Vonk W, Schriefers H 2002 Processing coordinated structures in context: the effect of topic-structure on ambiguity resolution. *J. Mem. Lang.* 46, 99–119. (doi:10.1006/jmla.2001.2800)

[21] Hoeks JCJ, Hendriks P, Vonk W, Brown CM, Hagoort P 2006 Processing the noun phrase versus sentence coordination ambiguity: thematic information does not completely eliminate processing difficulty. *Q. J. Exp. Psychol.* 59, 1581–1599. (doi:10.1080/17470210500268982) 10.1080/17470210500268982 16873110

[22] Bigand E, Pineau M 1997 Global context effects on musical expectancy. *Percept. Psychophys.* 59, 1098–1107. (doi:10.3758/BF03205524) 9360482 10.3758/bf03205524

[23] Kerkhofs R, Vonk W, Schriefers H, Chwilla DJ 2008 Sentence processing in the visual and auditory modality: do comma and prosodic break have parallel functions? *Brain Res.* 1224, 102–118. (doi:10.1016/j.brainres.2008.05.034) 18614156 10.1016/j.brainres.2008.05.034

[24] Groth-Marnat G 2001 The Wechsler intelligence scales. In *Specific learning disabilities and difficulties in children and adolescent: psychological assessment and evaluation*, pp. 29–52. New York, NY: Cambridge University Press (doi:10.1017/CBO9780511526794.003)

[25] Makuuchi M, Bahlmann J, Anwander A, Friederici AD 2009 Segregating the core computational faculty of human language from working memory. *Proc. Natl Acad. Sci. USA* 106, 8362–8367. (doi:10.1073/pnas.0810928106) 19416819 10.1073/pnas.0810928106 PMC2688876

[26] Fiebach CJ, Schlesewsky M, Lohmann G, von Cramon DY, Friederici AD 2005 Revisiting the role of Broca’s area in sentence processing: syntactic integration versus syntactic working memory. *Hum. Brain Mapp.* 24, 79–91. (doi:10.1002/hbm.20070) 15455462 10.1002/hbm.20070 PMC6871727

[27] Martin RC 1993 Short-term memory and sentence processing: evidence from neuropsychology. *Mem. Cogn.* 21, 176–183. (doi:10.3758/BF03202730) 10.3758/bf03202730 8469126

[28] Waters G, Caplan D, Alpert N, Stanczak L 2003 Individual differences in rCBF correlates of syntactic processing in sentence comprehension: effects of working memory and speed of processing. *NeuroImage* 19, 101–112. (doi:10.1016/S1053-8119(03)00007-7) 12781730 10.1016/s1053-8119(03)00007-7

[29] Ferreira F, Bailey KGD, Ferraro V 2002 Good-enough representations in language comprehension. *Curr. Dir. Psychol. Sci.* 11, 11–15. (doi:10.1111/1467-8721.00158)

[30] Featherstone CR, Morrison CM, Waterman MG, MacGregor LJ 2013 Semantics, syntax or neither? A case for resolution in the interpretation of N500 and P600 responses to harmonic incongruities. *PLoS ONE* 8, e76600 (doi:10.1371/journal.pone.0076600) 24223704 10.1371/journal.pone.0076600 PMC3818369

[31] Besson M, Faïta F, Peretz I, Bonnel A-M, Requin J 1998 Singing in the brain: independence of lyrics and tunes. *Psychol. Sci.* 9, 494–498. (doi:10.1111/1467-9280.00091)

[32] Carrus E, Pearce MT, Bhattacharya J 2013 Melodic pitch expectation interacts with neural responses to syntactic but not semantic violations. *Cortex* 49, 2186–2200. (doi:10.1016/j.cortex.2012.08.024) 23141867 10.1016/j.cortex.2012.08.024

[33] Fiveash A, Pammer K 2014 Music and language: do they draw on similar syntactic working memory resources? *Psychol. Music* 42, 190–209. (doi:10.1177/0305735612463949)

[34] Hagoort P 2005 On Broca, brain, and binding: a new framework. *Trends Cognit. Sci.* 9, 416–423. (doi:10.1016/j.tics.2005.07.004) 16054419 10.1016/j.tics.2005.07.004

[35] Rogalsky C, Rong F, Saberi K, Hickok G 2011 Functional anatomy of language and music perception: temporal and structural factors investigated using functional magnetic resonance imaging. *J. Neurosci.* 31, 3843–3852. (doi:10.1523/JNEUROSCI.4515-10.2011) 21389239 10.1523/JNEUROSCI.4515-10.2011 PMC3066175

