# Supplementary material for: Language influences music harmony perception: effects of shared syntactic integration resources beyond attention
Source: R Soc Open Sci. 2016 Feb 3;3(2):150685. doi: 10.1098/rsos.150685 (PMC4785990; doi:10.1098/rsos.150685)
Supplement: Kunert, Willems, & Hagoort, accepted_RSOS_supplementary material_v.4.0.docx Detailed analyses of Experiment 1's pretest, Experiment 1's analysis including the intermediate difficulty condition, Experiment 2's pre-tests, Experiment 1's ANCOVA analysis with working memory as a co-variate, combined ana [file rsos150685supp1.docx]

**Language influences music perception –**

**effects of shared syntactic integration resources beyond attention**

**Supplementary Materials**

Richard Kunert ^a,b, †^

Roel M. Willems ^a,b^

Peter Hagoort ^a,b^

^a^ Max Planck Institute for Psycholinguistics, P.O. Box 310, 6500 AH Nijmegen, The Netherlands

^b^ Radboud University Nijmegen, Donders Institute for Brain, Cognition and Behavior, P.O. Box 9010, 6500 GL Nijmegen, The Netherlands

^†^ Corresponding author: RiKunert@gmail.com

*2. Exp. 1*

*2.1.3. Pre-test: the strength of the language syntax and arithmetic manipulations*

Before starting the main experiment we conducted a pre-test with 24 participants who did not take part in experiment 1 (native Dutch speakers, age: *M* = 24.5, *SD* = 7.4, musical training: *M* = 4 years, *SD* = 3.8). The aim was to establish the strength of the difficulty manipulations in the language and the arithmetic tasks. Stimuli were presented as shown for the main experiment with three differences: 1) each trial was followed by a prompt, 2) there was no musical task and music was not presented, 3) after each trial participants had to rate the overall trial difficulty on a seven point Likert scale (1 = very easy, 7 = very difficult).

For the analysis of the language task, a one factor three-level (ambiguous S-coordination, unambiguous S-coordination, NP-coordination) ANOVA of the critical trials’ difficulty ratings showed a significant main effect [*F*_(2,46)_ = 14.08, *p* < .001, _p_η^2^ = .380]. Follow-up *t*-tests (Bonferroni corrected) revealed that the ambiguous S-coordination sentences were rated as significantly more difficult (*M* = 2.99, *SD* = 1.02) than the NP-coordination sentences (*M* = 2.46, *SD* = 1.11) [*t*_(23)_ = 4.67, *p* < .001, _p_η^2^ = .487]. The unambiguous S-coordination sentences received intermediate difficulty ratings (*M* = 2.90, *SD* = 1.03) which were not significantly lower than those for ambiguous S-coordinations [*t*_(23)_ < 1] but significantly higher than those for NP-coordinations [*t*_(23)_ = 4.51, *p* < .001, _p_η^2^ = .469]. Thus, the pre-test revealed that the syntactic language manipulation was generally noticeable with an indication that S-coordinations are more difficult in general, even when disambiguated by a comma.

For the analysis of the arithmetic task, a *t*-test revealed a significant difficulty effect (hard: *M* = 3.90, *SD* = 1.35; easy: *M* = 2.99, *SD* = 1.43) [*t*_(23)_ = 5.71, *p* < .001, _p_η^2^ = .586]. In order to compare the effects in the language and the arithmetic tasks we conducted an overall ANOVA with two factors: Task (language, arithmetic) and Difficulty (ambiguous S-coordination/hard, NP-coordination/easy). The arithmetic task was perceived as harder (*M* = 3.45, *SD* = 1.34) than the language task (*M* = 2.72, *SD* = 1.03) [Task, *F*_(1,23)_ = 14.48, *p* = .001, _p_η^2^ = .386]. Furthermore, ambiguous S-coordination/hard trials were generally rated as more difficult (*M* = 3.44, *SD* = 1.10) than NP-coordination/easy trials (*M* = 2.73, *SD* = 1.16) [Difficulty, *F*_(1,23)_ = 41.59, *p* < .001, _p_η^2^ = .644]. However, this difficulty effect was stronger in arithmetic than language, as revealed by a significant interaction effect [*F*_(1,23)_ = 5.47, *p* = .028, _p_η^2^ = .192].

*2.2.2.1. Critical language results including unambiguous S-coordination trials*

Unambiguous S-coordination trials, which include a disambiguating comma after the conjunction ‘**en**’ (***and***), were analysed alongside ambiguous S-coordination trials, which are identical to unambiguous S-coordination trials except for the absence of a comma, and NP-coordination trials. We used a 3 (Difficulty: ambiguous S-coordination, unambiguous S-coordination, NP-coordination) × 2 (Key: first key ending, second key ending) within-subjects ANOVA, see Figure S1. There was a difficulty main effect [*F*_(2,106)_ = 5.84, *p* = .004, _p_η^2^ = .099]. Follow-up *t*-tests (Bonferroni corrected) revealed that the garden-path sentences (ambiguous S-coordination) led to fewer high closure ratings (*M* = .65, *SD* = .18) than non-garden-path sentences (NP-coordination) (*M* = .72, *SD* = .15) [*t*_(53)_ = 3.46, *p* = .003, _p_η^2^ = .184]. The unambiguous S-coordination sentences were intermediate in this respect (*M* = .69, *SD* = .16), i.e. not significantly higher than the ambiguous S-coordinations [*t*_(53)_ = 1.69, *p* = .294, _p_η^2^ = .051] and not significantly lower than the NP-coordinations [*t*_(53)_ = 1.72, *p* = .276, _p_η^2^ = .053]. Otherwise, first key endings were less likely (*M* = .60, *SD* = .19) to receive a high closure rating than second-key ratings (*M* = .77, *SD* = .13) [Key, *F*_(1,53)_ = 75.64, *p* < .001, _p_η^2^ = .588]. These two factors did not interact [Key × Difficulty, *F*_(2,106)_ < 1].


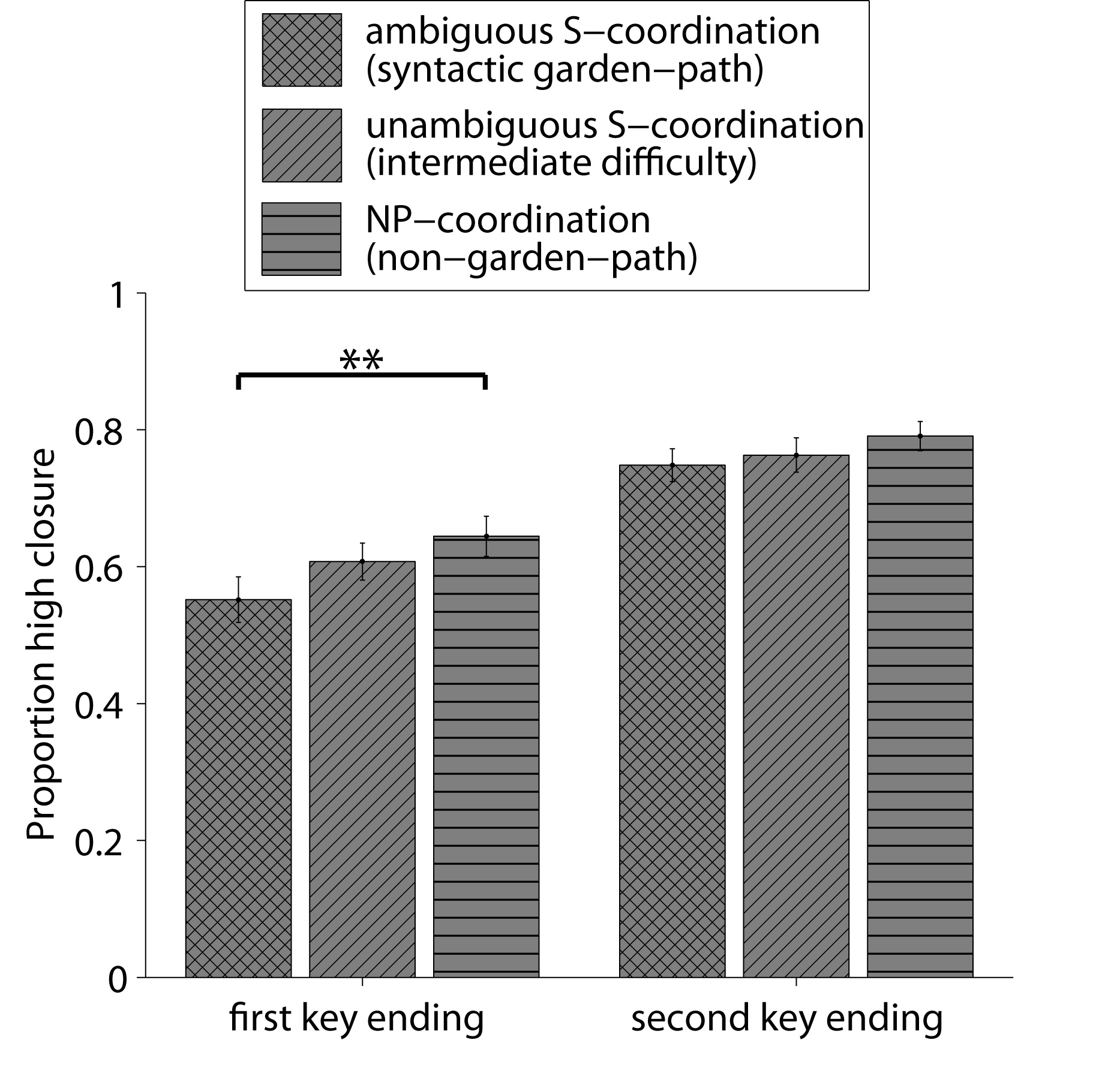


**Figure S1. Experiment 1: Closure ratings of critical language trials including intermediate difficulty condition.** Error bars = SEM. ** p < .01

*2.3. Analysis of language effect with z-scored digit span as co-variate*

The critical closure data of the musico-linguistic part of the experiment were analyzed in 2 (Difficulty: ambiguous S-coordination, NP-coordination) × 2 (Key: first key ending, second key ending) ANCOVAs with *z*-scored digit span as the co-variate. Before standardization, the spread in the digit span data was deemed sufficient to warrant this analysis (overall digit span: *M* = 16.78, *SD* = 3.39; forward digit span: *M* = 8.56, *SD* = 1.88; backward digit span: *M* = 8.20, *SD* = 2.29). Using overall digit span as the co-variate revealed the previously seen effects of Difficulty [*F*_(1,52)_ = 11.76, *p* = .001, _p_η^2^ = .184] and Key [*F*_(1,52)_ = 50.77, *p* < .001, _p_η^2^ = .494]. These two factors did not interact [Key × Difficulty, *F*_(1,52)_ = 1.82, *p* = .184, _p_η^2^ = .034]. None of these effects interacted with overall digit span [Difficulty × Digit Span, *F*_(1,52)_ < 1] [Key × Digit Span, *F*_(1,52)_ < 1] [Key × Difficulty × Digit Span, *F*_(1,52)_ = 1.45, *p* = .234, _p_η^2^ = .027].

Using forward digit span as the co-variate similarly revealed the previously seen effects of Difficulty [*F*_(1,52)_ = 12.31, *p* = .001, _p_η^2^ = .191] and Key [*F*_(1,52)_ = 50.95, *p* < .001, _p_η^2^ = .495]. These two factors did not interact [Key × Difficulty, *F*_(1,52)_ = 1.81, *p* = .184, _p_η^2^ = .034]. None of these effects interacted with forward digit span [Difficulty × Digit Span, *F*_(1,52)_ = 2.44, *p* = .124, _p_η^2^ = .045] [Key × Digit Span, *F*_(1,52)_ < 1] [Key × Difficulty × Digit Span, *F*_(1,52)_ = 1.32, *p* = .255, _p_η^2^ = .025].

Using backward digit span as the co-variate similarly revealed the previously seen effects of Difficulty [*F*_(1,52)_ = 12.07, *p* = .001, _p_η^2^ = .188] and Key [*F*_(1,52)_ = 50.91, *p* < .001, _p_η^2^ = .495]. These two factors did not interact [Key × Difficulty, *F*_(1,52)_ = 1.79, *p* = .187, _p_η^2^ = .033]. None of these effects interacted with backward digit span [Difficulty × Digit Span, *F*_(1,52)_ = 1.34, *p* = .252, _p_η^2^ = .025] [Key × Digit Span, *F*_(1,52)_ < 1] [Key × Difficulty × Digit Span, *F*_(1,52)_ < 1].

*3. Exp. 2*

*3.1.3. Pre-test 1: sentence completions show the intended misinterpretation of semantic material*

In a first pre-test we tested 54 participants who did not take part in experiment 2 (native Dutch speakers, age: *M* = 21.7, *SD* = 3.3, musical training: *M* = 4.2 years, *SD* = 4.1). Their task was to complete sentence beginnings (words one to eight, e.g., ‘De programmeur liet zijn **muis** op de tafel …’, *The programmer let his* ***mouse*** *…*) based on semantic items or filler items. Sentence beginnings were presented fully while the subject typed in a possible sentence ending. Half the sentence beginnings were based on filler items. Subsequently, sentence completions were rated independently by two blind raters (both Dutch native speakers) as to the interpretation of the manipulated word (‘**muis**’/’**veldmuis**’, ***mouse***/***field vole***: sentence completion indicative of an animal or not?). Many sentence completions (22%) were not rateable according to the raters (uninformative or absent sentence completions) and the two raters sometimes (18% or trials) disagreed about this. When both agreed that an item was rateable their agreement was 99.4%. Any disagreements were resolved by a third blind rater (also a Dutch native speaker). Garden-path and non-garden-path sentence beginnings did not differ according to the rateability of the completions [*t*_(53)_ < 1]. However, using the rateable trials only, the intended word interpretation was overwhelmingly adopted in the non-garden-path condition (93.6%) while this hardly happened in the semantic garden-path condition (13.3%) [*t*_(53)_ = 52.18, *p* < .001, _p_η^2^ = .981].

*3.1.4. Pre-test 2: the strength of the syntactic and semantic manipulations*

The aim of the second pre-test was to establish the strength of the difficulty manipulations of the syntactic and the semantic items. We tested 24 participants who did not take part in the first pre-test or the main experiment (native Dutch speakers, age: *M* = 21.8, *SD* = 3.1, musical training: *M* = 4 years, *SD* = 4.2). Stimuli were presented as shown for the main experiment with five differences: 1) the length of word presentation was controlled by the participant (self-paced reading) giving us an online measure of processing difficulty, 2) each trial was followed by a prompt, 3) there was no musical task and music was not presented, 4) after each sentence participants had to rate the acceptability of the sentence (data not presented here), 5) after each trial participants had to rate the overall trial difficulty on a seven point Likert scale (1 = very easy, 7 = very difficult).

For the analysis of the language syntax part, we first analysed difficulty ratings. As expected, object-relative clause trials were rated as more difficult (*M* = 3.24, *SD* = 0.93) than subject-relative clauses (*M* = 2.89, *SD* = 0.80) [*t*_(23)_ = 3.96, *p* = .001, _p_η^2^ = .405]. In order to analyse the reading time data we took the natural logarithm of reading times and defined a trial as an outlier if it fulfilled any of the following three criteria: (1) shorter than 50ms, (2) longer than 2500ms, (3) longer than 2.5 *SD* above the sentence-position-specific mean (only defined as an outlier if this criterion is reached both in analysis by items and by subjects). Outlier values (3.13% of all reading times, 5.98% of critical word reading times) were replaced by the cut-off value. The eighth word was the pre-critical word and as expected it did not show a significant difference between syntax conditions (OR: *M* = 509 ms; SR: *M* = 505 ms)^[[1]](#footnote-1)^ [*t*_(23)_ < 1]. However, both the critical ninth word [*t*_(23)_ = 2.83, *p* = .009, _p_η^2^ = .259] and the post-critical tenth word [*t*_(23)_ = 2.91, *p* = .008, _p_η^2^ = .269] were read longer in the object-relative clause condition (critical: *M* = 723 ms; post-critical: *M* = 665 ms) than in the subject-relative clause condition (critical: *M* = 659 ms; post-critical: *M* = 607 ms).

Carrying out the same analyses for the language semantics part, we also found a significant effect of semantic manipulation on trial difficulty ratings [*t*_(23)_ = 3.33, *p* = .003, _p_η^2^ = .325], indicating that semantic garden-path sentences were perceived as more difficult (*M* = 2.58, *SD* = 0.90) than the non-garden-path sentences (*M* = 2.26, *SD* = 0.74). The reading time analysis did not reveal a significant difference between conditions on the pre-critical word (GP: *M* = 413 ms; non-GP: *M* = 410 ms) [*t*_(23)_ < 1], while for both the critical word [*t*_(23)_ = 3.12, *p* = .005, _p_η^2^ = .297] as well as the post-critical word [*t*_(23)_ = 4.64, *p* < .001, _p_η^2^ = .484] the same words were read longer in the semantic garden-path condition (critical: *M* = 526 ms; post-critical: *M* = 533 ms) than in the non-garden-path condition (critical: *M* = 482 ms; post-critical: *M* = 448 ms).

In order to compare the effects of the syntax and the semantics manipulations we conducted ANOVAs with two factors: Manipulation (syntax, semantics) and Difficulty (object-relative clause/semantic garden-path, subject-relative clause/non-garden-path). The trial difficulty ratings showed that syntax trials were generally perceived as harder (*M* = 3.06, *SD* = 0.84) than semantics trials (*M* = 2.42; *SD* = 0.79) [Manipulation, *F*_(1,23)_ = 20.39, *p* < .001, _p_η^2^ = .470] and syntactically or semantically more challenging trials were rated as more difficult (*M* = 2.91, *SD* = 0.81) than less challenging trials (*M* = 2.57, *SD* = 0.69) [Difficulty, *F*_(1,23)_ = 23.73, *p* < .001, _p_η^2^ = .508]. However, crucially, these two factors did not interact [*F*_(1,23)_ < 1]. The pattern in the reading time data is similar. For the pre-critical word reading time analysis, words in the syntax part were read longer (*M* = 507 ms) than in the semantics part (*M* = 411 ms) [Manipulation, *F*_(1,23)_ = 25.75, *p* < .001, _p_η^2^ = .528]. As expected, the difficulty effect as well as the interaction were non-significant [Difficulty, *F*_(1,23)_ < 1] [Manipulation × Difficulty, *F*_(1,23)_ < 1]. For the critical word reading time analysis, words in the syntax part were still read longer (syntax: *M* = 690 ms; semantics: *M* = 504 ms) [Manipulation, *F*_(1,23)_ = 18.85, *p* < .001, _p_η^2^ = .450]. Also, words in more challenging sentences were read longer (OR/GP: *M* = 616 ms; SR/non-GP: *M* = 564 ms) [Difficulty, *F*_(1,23)_ = 15.77, *p* = .001, _p_η^2^ = .407]. However, these two factors did not interact [Manipulation × Difficulty, *F*_(1,23)_ < 1]. For the post-critical word, words in the syntactic part were still read longer (syntax: *M* = 636 ms; semantics: *M* = 489 ms) [Manipulation, *F*_(1,23)_ = 27.27, *p* < .001, _p_η^2^ = .542]. Furthermore, there was an effect of difficulty (OR/GP: *M* = 596 ms; SR/non-GP: *M* = 521 ms) [*F*_(1,23)_ = 27.39, *p* < .001, _p_η^2^ = .544]. These two factors marginally interacted [Manipulation × Difficulty, *F*_(1,23)_ = 3.05, *p* = .094, _p_η^2^ = .117] indicating that the semantic garden-path effect was slightly greater than the syntax effect in the reading times of the post-critical word.

*4.1.1. Combined analysis of syntax effect in experiments 1 and 2 with musical training as co-variate*

A 2 (Experiment: one, two) × 2 (Difficulty: ambiguous S-coordination/object-relative clause, NP-coordination/subject-relative clause) × 2 (Key: first key ending, second key ending) mixed between- and within-subjects ANCOVA with *z*-scored musical training as a co-variate exhibited the same significant effects seen in the equivalent ANOVA without a co-variate [Difficulty, *F*_(1,113)_ = 17.47, *p* < .001, _p_η^2^ = .134] [Key, *F*_(1,113)_ = 89.36, *p* < .001, _p_η^2^ = .442] [Difficulty × Key, *F*_(1,113)_ = 4.19, *p* = .043, _p_η^2^ = .036]. The factor experiment was still without effect [Experiment, *F*_(1,113)_ < 1] [Difficulty × Experiment, *F*_(1,113)_ = 1.38, *p* = .243, _p_η^2^ = .012] [Key × Experiment, *F*_(1,113)_ < 1] [Difficulty × Key × Experiment, *F*_(1,113)_ < 1]. Musical training did not modulate any of these effects [Musical Training, *F*_(1,113)_ = 1.19, *p* = .278, _p_η^2^ = .010] [Difficulty × Musical Training, *F*_(1,113)_ < 1] [Key × Musical Training, *F*_(1,113)_ = 1.05, *p* = .307, _p_η^2^ = .009] [Difficulty × Key × Musical Training, *F*_(1,113)_ = 1.61, *p* = .207, _p_η^2^ = .014].

*4.1.2. Results by items*

In order to check whether the syntax effect does not only generalise across subjects but also across items, we further analysed the data with linguistic items (*F*2-analysis) and musical items (*F*3-analysis) as random factors. That is, we averaged music ratings not by participants, but instead across participants by items.

*Experiment 1*. Two 2 (Task: language, arithmetic) × 2 (Difficulty: ambiguous S-coordination/hard, NP-coordination/easy) × 2 (Key: first key ending, second key ending) ANOVAs were performed. Task is a between-items factor in the analysis by linguistic items (*F*2-analysis) but a within-items factor in the analysis by musical items (*F*3-analysis). All other factors were within-items in both ANOVAs. The analyses exhibited a significantly greater difficulty effect in the language task than the arithmetic task by linguistic items only [Task × Difficulty, *F*2_(1,98)_ = 6.25, *p* = .014, _p_η^2^ = .060; *F*3_(1,9)_ = 3.58, *p* = .091, _p_η^2^ = .284]. Otherwise, the Task factor showed a main effect by musical items only [Task, *F*2_(1,98)_ = 3.24, *p* = .075, _p_η^2^ = .032; *F*3_(1,9)_ = 15.25, *p* = .004, _p_η^2^ = .629], while the other two main effects were significant by linguistic and musical items [Difficulty, *F*2_(1,98)_ = 10.32, *p* = .002, _p_η^2^ = .095; *F*3_(1,9)_ = 5.38, *p* = .046, _p_η^2^ = .374] [Key, *F*2_(1,98)_ = 40.50, *p* < .001, _p_η^2^ = .292; *F*3_(1,9)_ = 19.41, *p* = .002, _p_η^2^ = .683]. The Key and Task factors interacted by musical items only [*F*2_(1,98)_ = 2.57, *p* = .112, _p_η^2^ = .026; *F*3_(1,9)_ = 9.14, *p* = .014, _p_η^2^ = .504] while Key and Difficulty did not [*F*2_(1,98)_ < 1; *F*3_(1,9)_ < 1]. The three-way interaction was not significant [Task × Difficulty × Key, *F*2_(1,98)_ < 1; *F*3_(1,9)_ = 1.33, *p* = .278, _p_η^2^ = .129].

*Experiment 2*. Two 2 (Manipulation: syntax, semantics) × 2 (Difficulty: object-relative clause/semantic garden-path, subject-relative clause/non-garden-path) × 2 (Key: first key ending, second key ending) ANOVAs were performed. As in experiment 1, Task is a between-items factor in the analysis by linguistic items (*F*2-analysis) but a within-items factor in the analysis by musical items (*F*3-analysis). All other factors were within-items in both ANOVAs. The analyses exhibited a significantly greater difficulty effect in the syntax part than in the semantics part by musical items only [Manipulation × Difficulty, *F*2_(1,78)_ = 2.71, *p* = .103, _p_η^2^ = .034; *F*3_(1,9)_ = 15.07, *p* = .004, _p_η^2^ = .626]. Otherwise, two of the three factors showed a main effect [Manipulation, *F*2_(1,78)_ = 6.57, *p* = .012, _p_η^2^ = .078; *F*3_(1,9)_ = 5.71, *p* = .041, _p_η^2^ = .388] [Difficulty, *F*2_(1,78)_ < 1; *F*3_(1,9)_ < 1] [Key, *F*2_(1,78)_ = 132.78, *p* < .001, _p_η^2^ = .630; *F*3_(1,9)_ = 10.70, *p* = .010, _p_η^2^ = .543]. The Key and Manipulation factors did not interact [*F*2_(1,78)_ < 1; *F*3_(1,9)_ < 1], neither did the Key and Difficulty factors [*F*2_(1,78)_ = 1.35, *p* = .250, _p_η^2^ = .017; *F*3_(1,9)_ < 1]. The three-way interaction was not significant either [Manipulation × Difficulty × Key, *F*2_(1,78)_ = 1.01, *p* = .317, _p_η^2^ = .013; *F*3_(1,9)_ = 1.89, *p* = .201, _p_η^2^ = .174].

*Experiments 1 and 2 combined*. Two 2 (Experiment: one, two) × 2 (Difficulty: ambiguous S-coordination/object-relative clause, NP-coordination/subject-relative clause) × 2 (Key: first key ending, second key ending) ANOVAs were performed. Experiment is a between-items factor in the analysis by linguistic items (*F*2-analysis) but a within-items factor in the analysis by musical items (*F*3-analysis). All other factors were within-items in both ANOVAs. The analyses exhibited the main effects of Difficulty and Key which we observed before [Difficulty, *F*2_(1,98)_ = 10.81, *p* = .001, _p_η^2^ = .099; *F*3_(1,9)_ = 10.63, *p* = .010, _p_η^2^ = .541] [Key, *F*2_(1,98)_ = 82.09, *p* < .001, _p_η^2^ = .456; *F*3_(1,9)_ = 19.63, *p* = .002, _p_η^2^ = .686]. The factor Experiment was without effect [Experiment, *F*2_(1,98)_ = 2.25, *p* = .137, _p_η^2^ = .022; *F*3_(1,9)_ = 1.71, *p* = .223, _p_η^2^ = .160] [Difficulty × Experiment, *F*2_(1,98)_ = 1.05, *p* = .308, _p_η^2^ = .011; *F*3_(1,9)_ < 1] [Key × Experiment, *F*2_(1,98)_ < 1; *F*3_(1,9)_ = 1.12, *p* = .318, _p_η^2^ = .110] [Difficulty × Key × Experiment, *F*2_(1,98)_ < 1; *F*3_(1,9)_ < 1]. The Difficulty × Key interaction was significant by musical items only [*F*2_(1,98)_ = 1.87, *p* = .175, _p_η^2^ = .019; *F*3_(1,9)_ = 5.17, *p* = .049, _p_η^2^ = .365].

*6. Post-test: Closure ratings without concurrent task*

In order to check whether the double-task paradigm we chose led to unusual music ratings, we ran a post-test on the music material only. Sixty new participants (age: *M* = 23.1, *SD* = 4.4; musical training: *M* = 5.1 years, *SD* = 5.3) rated the closure of 80 music sequences, as done in each block of experiment 1 or experiment 2. However, there was no concurrent task in the visual modality. The procedure and stimuli were otherwise the same as in the experiments.

As can be seen in Figure S2, the general pattern in this post-test mirrors what is seen in the experiments. All participants gave more high closure ratings to authentic cadence endings than no-cadence endings (*M*_authentic cadence_ = .72 > *M*_no cadence_ = .09, difference *SD* = .63). There were also more high closure ratings for second-key endings (*M* = .60, *SD* = .15) than first-key endings (*M* = .56, *SD* = .15). However, as opposed to the results of experiments 1 and 2, this difference was not significant [*t*_(59)_ = 1.21, *p* = .232, _p_η^2^ = .024]. As shown in Figure S2, this likely reflects the unexpectedly low number of high closure ratings of second key endings, compared to experiments 1 and 2. The implications for the role of split attention on the ability to integrate chords into a new harmonic key are arguably beyond the scope of this paper. Here, we just want to point out that our claims on shared syntactic processing in music and language are based on first-key endings which appear unaffected by the difference between split-attention (experiments 1 and 2) and full attention (post-test).


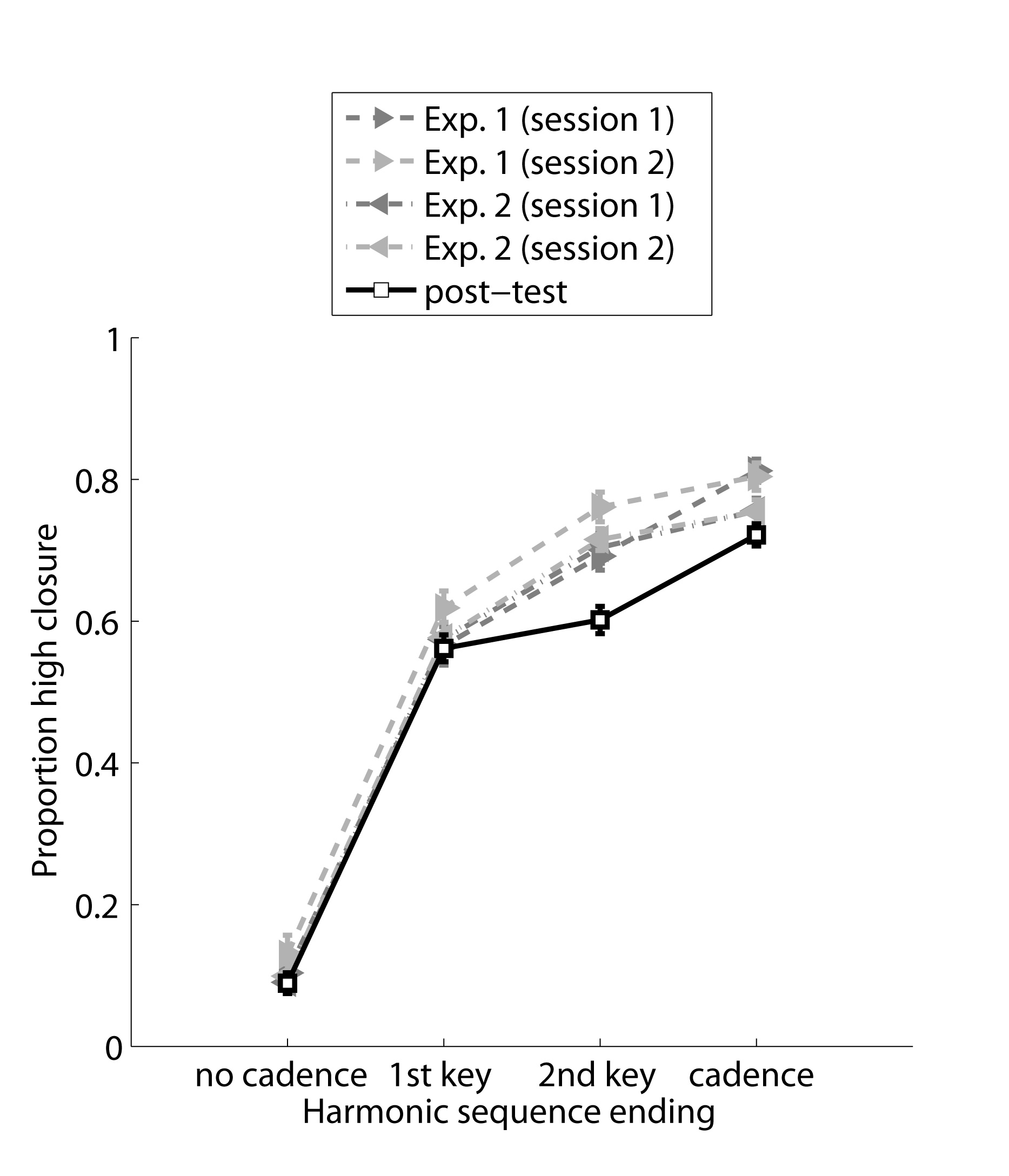


**Figure S2. Post-test: Closure ratings of music material without second task.** Error bars = SEM.

*7. Additional analyses of experiments 1 & 2: does task order influence the results?*

An anonymous reviewer asked whether the order of language syntax and control trials influenced the findings we present. It should be noted that task order was counter-balanced. Therefore, a systematic influence is very unlikely. Still, for the sake of completeness, we present the analysis of music closure ratings with the additional within-subjects factor Order.

The ANOVA of the language block of experiment 1 (see *section 2.2.2.1*) with the additional factor Order reveals the same significant effects as before (Difficulty, Key; *p*s < .01). The factor Order was not significant, neither as a main effect [*F*_(1,52)_ = 3.41, *p* = .071, _p_η^2^ = .061], nor as an interaction with any other effect (*p*s > .1). The ANOVA of the syntax block of experiment 2 (see *section 3.2.2.1*) with the additional factor Order reveals the same significant effects as before (Difficulty, Key; *p*s < .05). The factor Order was not significant, neither as a main effect [*F*_(1,60)_ < 1], nor as an interaction with any other effect (*p*s > .2).

We also carried out an ANOVA of the combined syntax data of experiments 1 and 2 with the factors Order (language syntax trials run first or second), Experiment (experiment 1, experiment 2), Difficulty (ambiguous S-coordination/object-relative clause, NP-coordination/subject-relative clause) and Key (first key ending, second key ending). The first two factors were between-subjects, the other two factors were within-subjects. The significant effects reported in the main analysis without the factor Order were still significant (Difficulty, Key, Difficulty × Key; *p*s < .05). The factor Order was marginally significant (*F*_(1,112)_ = 3.06, *p* = .083, _p_η^2^ = .027), indicating a slightly higher proportion of high closure ratings in trials run after the control task (*M* = .695) compared to before (*M* = .650). No other effect was significant (*p*s > .2). This shows that task order did not interact with any of the effects of interest.

1. For ease of interpretation we report the mean logged values as *e*^ln(value)^, i.e. de-logged into standard milliseconds. [↑](#footnote-ref-1)
